# Mn-Mimochrome VI^*^a: An Artificial Metalloenzyme With Peroxygenase Activity

**DOI:** 10.3389/fchem.2018.00590

**Published:** 2018-12-04

**Authors:** Linda Leone, Daniele D'Alonzo, Véronique Balland, Gerardo Zambrano, Marco Chino, Flavia Nastri, Ornella Maglio, Vincenzo Pavone, Angela Lombardi

**Affiliations:** ^1^Department of Chemical Sciences, University of Naples “Federico II”, Naples, Italy; ^2^Laboratoire d'Electrochimie Moléculaire, UMR 7591 CNRS, Université Paris Diderot, Sorbonne Paris Cité, Paris, France; ^3^Institute of Biostructures and Bioimages, National Research Council, Naples, Italy

**Keywords:** artificial metalloenzymes, biocatalysis, manganese porphyrins, oxidation catalysis, heme-protein models

## Abstract

Manganese-porphyrins are important tools in catalysis, due to their capability to promote a wide variety of synthetically valuable transformations. Despite their great reactivity, the difficulties to control the reaction selectivity and to protect the catalyst from self-degradation hamper their practical application. Compared to small-molecule porphyrin complexes, metalloenzymes display remarkable features, because the reactivity of the metal center is finely modulated by a complex interplay of interactions within the protein matrix. In the effort to combine the catalytic potential of manganese porphyrins with the unique properties of biological catalysts, artificial metalloenzymes have been reported, mainly by incorporation of manganese-porphyrins into native protein scaffolds. Here we describe the spectroscopic and catalytic properties of Mn-Mimochrome VI^*^a (Mn-MC6^*^a), a mini-protein with a manganese deuteroporphyrin active site within a scaffold of two synthetic peptides covalently bound to the porphyrin. Mn-MC6^*^a is an efficient catalyst endowed with peroxygenase activity. The UV-vis absorption spectrum of Mn-MC6^*^a resembles that of Mn-reconstituted horseradish peroxidase (Mn-HRP), both in the resting and high-valent oxidized states. Remarkably, Mn-MC6^*^a shows a higher reactivity compared to Mn-HRP, because higher yields and chemoselectivity were observed in thioether oxidation. Experimental evidences also provided indications on the nature of the high-valent reactive intermediate and on the sulfoxidation mechanism.

## Introduction

Nature mastered coordination chemistry in a fascinating manner, as proven by the remarkable features of metalloenzymes (Wolfenden and Snider, 2001; Valdez et al., 2014). To take full advantage of the rich chemistry of metal ions, in terms of spectroscopic, magnetic, and catalytic properties, proteins have evolved as complex macromolecular ligands. Indeed, the protein matrix exerts a fine control on the reactivity of metal ions, through a variety of interactions, ranging from coordinate and hydrogen bond, to hydrophobic and ionic interactions (Ragsdale, 2006; Maglio et al., 2012). This control allows proteins to benefit from the redox and Lewis-acid catalysis of metal ions. As in a mutual relationship, metal ions themselves can make the protein functional. Thanks to their preferred coordination geometry, metal ions may act as templates, binding various domains of the protein together and bringing reactive groups in the correct relative orientation for activity (Maglio et al., 2012).

The strong interplay between metal cofactor and protein scaffold is finely exemplified by the functional versatility of heme-containing enzymes (Bowman and Bren, 2008; Poulos, 2014). Peroxidases, catalases and monooxygenases share, in their catalytic cycle, a similar high-valent iron–oxo intermediate, whose fate depends on the specific environment created by the surrounding protein matrix (Dolphin et al., 1971; Hersleth et al., 2006; Moody and Raven, 2018).

Studies on small-molecule mimics, based on synthetic metallo-porphyrinoid complexes, gave basic insights into the nature of the intermediates and into the reaction mechanisms of heme-enzymes (Groves, 2006; Karlin, 2010; Baglia et al., 2017). Modification of metallo-porphyrins with different chemical moieties, assembled to resemble and mimic the protein matrix, clearly highlighted the environment influence over metal cofactor reactivity (Nastri et al., 1998; Fujii, 2002). Nevertheless, with small-molecule mimics is very difficult to obtain the environment complexity offered by the protein matrix in natural systems.

In the last two decades, a variety of approaches have been exploited to cage metallo-porphyrins into protein scaffolds of different complexity for the development of artificial heme-enzymes (Nastri et al., 2013; Chino et al., 2018). By mimicking Nature's strategy, directed evolution allowed repurposing heme-enzymes toward abiotic reactions (Arnold, 2018). Heme-protein redesign, through scaffold engineering and/or cofactor replacement, afforded new enzymes with a variety of functionalities (Garner et al., 2011; Cai et al., 2013; Oohora et al., 2017). Further, *de novo* design approaches afforded the construction of artificial peroxidases with impressive enzymatic rate constants (Patel and Hecht, 2012; D'Souza et al., 2017; Watkins et al., 2017).

Using a structure-based approach and a miniaturization process (Lombardi et al., 2000), we developed a class of heme-protein models named Mimochromes (Nastri et al., 1998; Lombardi et al., 2001). Mimochromes consist of two small peptide chains covalently linked to the deuteroporphyrin through amide bonds between the heme-propionic groups and the ε-amino groups of lysine residues. The peptide chains are conceived to embrace the porphyrin in a helix-heme-helix sandwiched structure, thus reproducing the protein environment found in natural systems.

Starting from the prototype molecule (Mimochrome I, herein referred as MC1) (Nastri et al., 1997), a redesign approach allowed to optimize the scaffold (Lombardi et al., 2003; Di Costanzo et al., 2004). Subsequent rounds of design were aimed at engineering functionality in the optimized scaffold. The overall process afforded the catalytically active derivative Mimochrome VI (MC6), which mimics the asymmetry of natural proteins in both primary and secondary coordination spheres (Ranieri et al., 2010; Nastri et al., 2011). MC6 is made up of a tetradecapeptide (*TD*) bearing the His axial ligand, and a decapeptide (*D*) lacking coordinating residue, which allows to create a substrate binding pocket on the distal side of the heme. These structural features steered Fe^III^-MC6 toward peroxidase catalysis.

**Figure 1:**
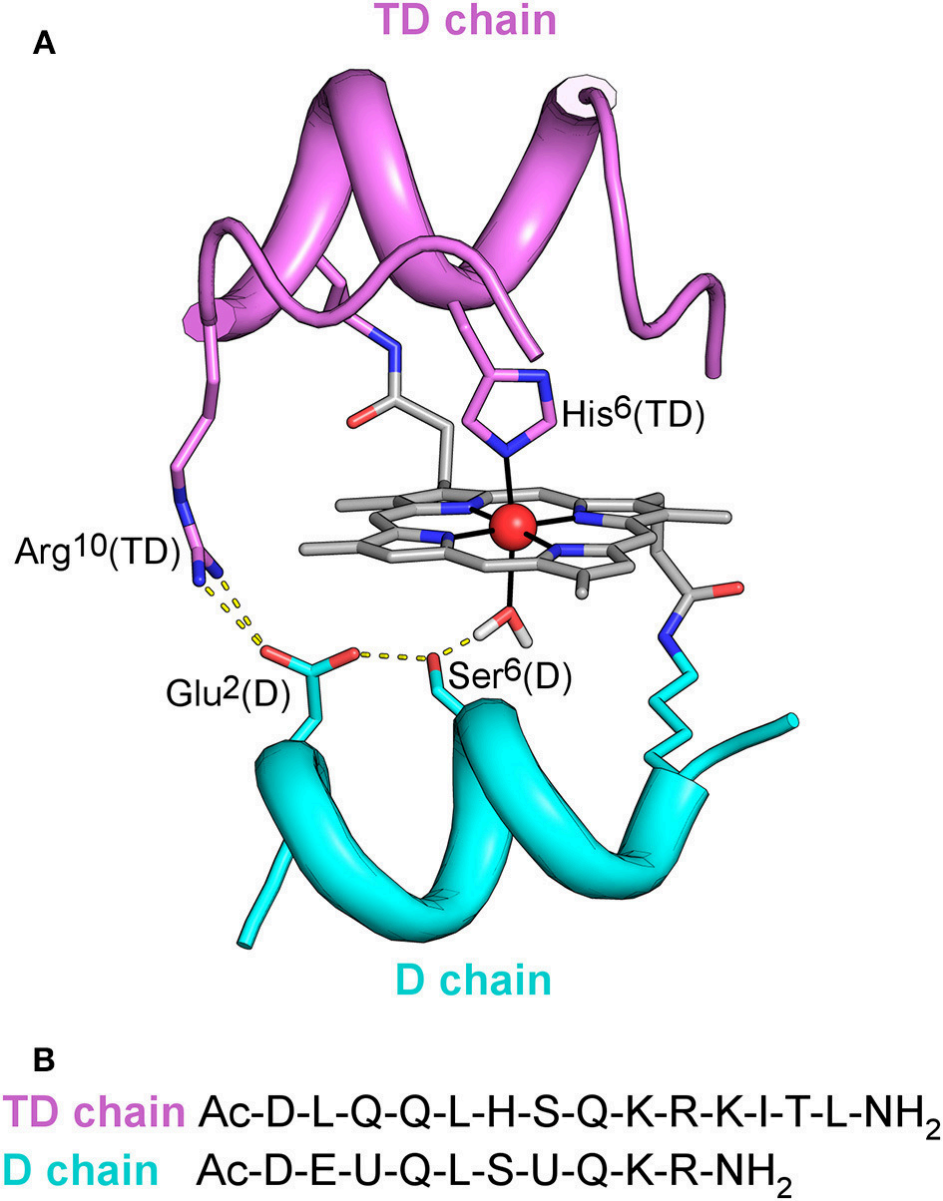
**(A)** MC6^*^a designed model. Backbone of peptide chains is depicted as ribbon. Side-chains of functional and structural residues are depicted as sticks, and the metal ion as red ball. The designed hydrogen bonding network, involving Arg^10^ in the *TD* chain, Asp^1^, Ser^6^ in the *D* chain, and the metal-bound water molecule is also highlighted. **(B)** Peptide sequences of *TD* and *D* peptide chains. U denotes the α,α-dimethyl glycine (Aib) residue.

The simplicity of the MC6 scaffold offered us the opportunity for structure/function relationship studies. The effects of second-shell interactions (Vitale et al., 2015) and of conformational constraints (Caserta et al., 2018) in tuning catalysis were systematically evaluated. Site-specific mutations in both peptide chains allowed to select MC6^*^a (Figure 1) as the best peroxidase catalyst among the mimochrome family. Fe^III^-MC6^*^a exceeds the turnover frequency and the total turnover number (TON) of its best predecessor, displaying a 20-fold higher catalytic efficiency compared to that of natural horseradish peroxidase (HRP) in oxidation of 2,2′-azino-bis(3-ethylbenzothiazoline-6-sulphonic acid) (ABTS) (Caserta et al., 2018). Moreover, the cobalt derivative (Co-MC6^*^a) behaves as a very promising catalyst in hydrogen evolution reactions, as it was able to electrocatalytically reduce protons to hydrogen (H_2_) in water at neutral pH under aerobic conditions, performing more than 230,000 turnovers (Firpo et al., 2018). Finally, we have also demonstrated that mimochromes can be successfully conjugated to gold nanoparticles (AuNPs) and/or immobilized onto electrode surfaces while preserving the redox properties and the peroxidase activity (Ranieri et al., 2010; Vitale et al., 2014; Zambrano et al., 2018).

The goal of the present work was to further evaluate the versatility of the MC6^*^a scaffold toward metal replacement, by swapping iron to manganese. Iron– and manganese–porphyrins have rich redox chemistry (Felton, 1978), as both metal ions have access to a wide range of oxidation and spin states. They also share a common metal-oxo species during catalysis, and the enhanced stability of Mn-oxo over the corresponding Fe-oxo species (Neu et al., 2015; Chino et al., 2018) has allowed to get deep insights into the nature of the active species and to shed light on their role in catalysis (Gelb et al., 1982; Nick et al., 2002). Further, the activation of Mn^III^ to the reactive Mn^IV^ or Mn^V^ species (Song et al., 2007; Neu et al., 2014) has been found to promote a variety of synthetically relevant reactions, ranging from the epoxidation (Srour et al., 2012) and sulfoxidation (Neu et al., 2014) up to the site-selective functionalization of unactivated C-H bonds (Martinez-Lorente et al., 1996; Costas, 2011; Liu et al., 2012; Liu and Groves, 2015).

Herein we report the synthesis and spectroscopic characterization of Mn-MC6^*^a, and of its high-valent Mn-oxo species. The ability of this species in promoting the oxy-functionalization of reducing substrates was also evaluated and compared with that of Fe-MC6^*^a. Both iron and manganese complexes showed peroxygenase activity, thus highlighting that MC6^*^a scaffold is able to host both metal-oxo species and tune their reactivity.

## Results and Discussion

### Synthesis, Purification, and Analysis

**Figure 2:**
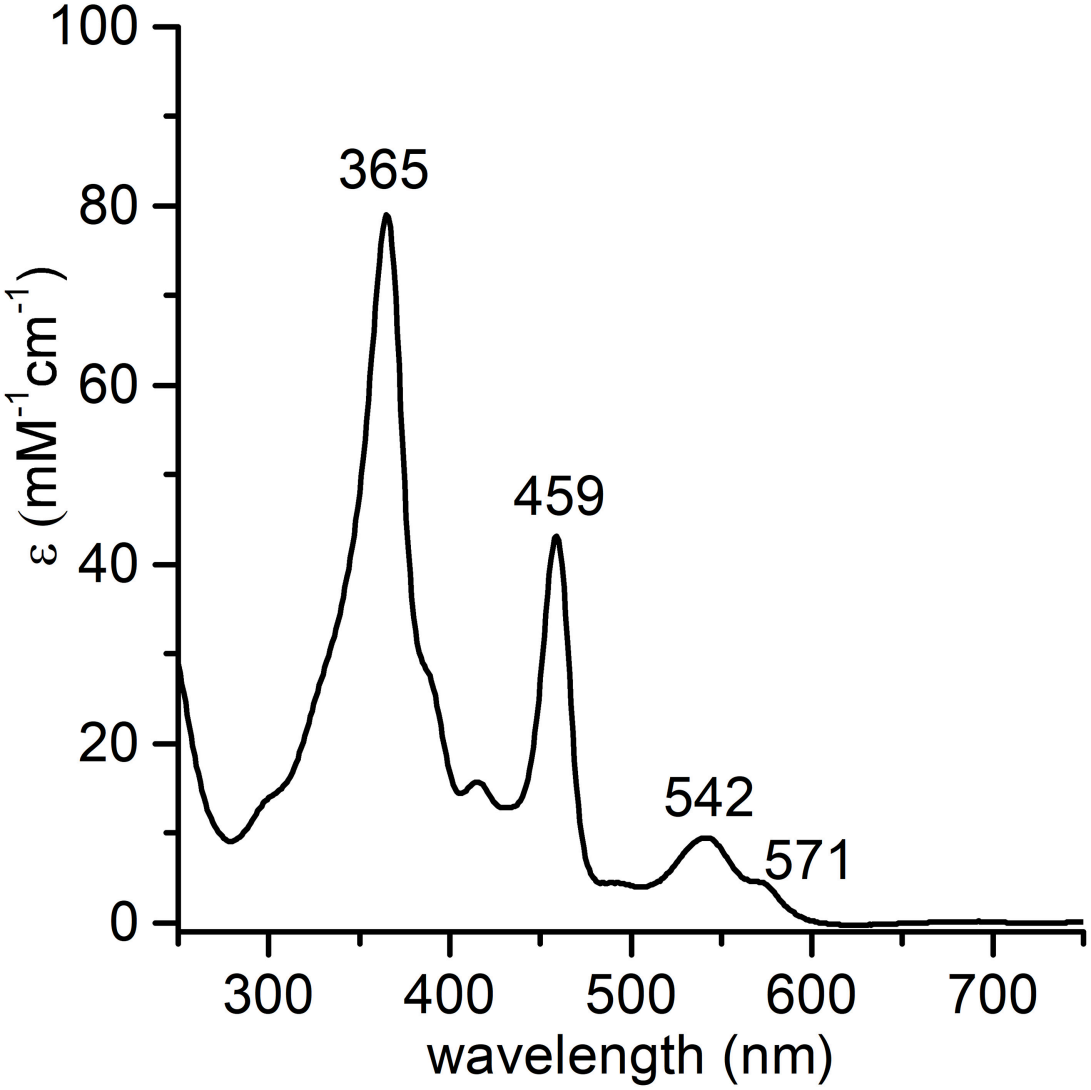
UV-vis absorption spectrum of Mn^III^-MC6^*^a (1.0·10^−5^ M) in acid aqueous solution (0.1 % TFA *v/v*, pH 1.8).

The synthesis and purification of *apo*-MC6^*^a and Fe-MC6^*^a were carried out using previously described procedures (Caserta et al., 2018). The manganese ion insertion was successfully carried out by following a variation of literature methods (Caserta et al., 2018) using Mn(OAc)_2_ as the metal source (Dolphin, 1978). LC-MS analysis confirmed manganese ion insertion into *apo*-MC6^*^a (Figure S1). Deconvolution analysis of the positive ESI-MS mass spectrum gave an experimental mass of 3489.2 ± 0.4 Da, in agreement with the theoretical value (3488.8 Da). Product identity was further confirmed by UV-vis absorption spectroscopy (Figure 2). The UV-vis spectrum of Mn-MC6^*^a in acid aqueous solution (H_2_O 0.1% trifluoroacetic acid, TFA) shows a typical split Soret band (Boucher, 1968), with one component centered at 365 nm and a weaker component at 459 nm. The Q-band region is characterized by two bands at 542 nm (β) and 571 nm (α). Three additional weak absorption bands at 310, 390, 420 nm are also present. These spectral data are in good agreement with those reported for Mn-porphyrins, with a hexa-coordinated, high-spin Mn^III^ ion (Giovannetti et al., 2010). Mn-MC6^*^a stock solutions, analyzed for metal contents by atomic absorption spectroscopy, and properly diluted, enabled the calculation of a molar absorptivity of (7.86 ± 0.08) 10^4^ M^−1^ cm^−1^ at 365 nm.

### Spectroscopic and Electrochemical Characterization

UV-vis and circular dichroism (CD) spectroscopies were combined to gain information about the role of the 2,2,2-trifluoroethanol (TFE) and pH on the Mn^III^-MC6^*^a structural and coordination properties. Electrochemical analysis was also performed to get further insight into the metal ion coordination states at different pHs.

#### Structural Properties of Mn^III^-MC6^*^a by CD Spectroscopy

**Figure 3:**
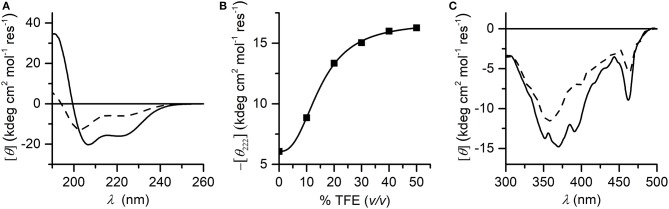
**(A)** Far-UV CD spectra of Mn-MC6^*^a (2.0·10^−5^ M) acquired in 5 mM phosphate buffered solution (pH 6.5) in absence (dashed line) and in presence (plain line) of 40% TFE (*v/v*); **(B)** titration curve showing –[θ_222_] as a function of TFE concentration (% *v/v*). **(C)** CD spectra in the Soret-band region acquired in the same conditions of the panel **(A)**.

CD spectroscopy was used to analyze the structural properties of Mn^III^-MC6^*^a. In particular, the role played by the helix-inducing solvent TFE on the structure was investigated (Hong et al., 1999; Vitale et al., 2015). To this end, far-UV CD spectra were acquired in 5 mM phosphate buffer solution at pH 6.5, at different TFE concentrations (in the range of 0–50% *v/v*) (Figure 3). Inspection of Figure 3A shows that in the absence of TFE the peptide chains are poorly structured, but reminiscent of an α-helix secondary structure (Whitmore and Wallace, 2008). Figure 3B shows the plot of θ_222_ as a function of TFE and Table 1 reports far-UV region CD parameters at 0 and 40% TFE (*v/v*). Addition of TFE contributes to enhance the α-helical content, as assessed by: (i) the increase of the mean residue ellipticity at 222 nm (θ_222_), (ii) the enhancement of the θ_ratio_ (θ_222_/θ_min_) that progressively approaches the unity, (iii) the shift of the lower minimum (λ_min_) toward 207 nm, (iv) the λ_0_ shift to higher wavelengths. Similarly to Fe^III^-MC6^*^a (Caserta et al., 2018), the maximum α-helical content was reached at 40% TFE (*v/v*).

**Table 1:** Far-UV region CD parameters of Mn-MC6^*^a in phosphate buffer/TFE solution pH 6.5.

**% TFE (*v/v*)**	**[θ_222_]^a^**	**[θ_min_]^a^, ^b^ (λ, nm)**	**θ${}_{\text{ratio}}^{\text{c}}$**	**$\lambda_{0}^{\text{d}}$ (nm)**
0	−6,072	−12,803 (203)	0.47	194
40	−16,266	−20,263 (207)	0.80	199

a *[θ] is expressed as mean residue ellipticity (deg cm^2^ dmol^-1^ res^−1^)*;

b *[θ_min_] represents the [θ] value at the shorter wavelength minimum (reported in parentheses); ${}^{\text{c}}\theta_{\text{ratio}}$ is the ratio [θ_222_]/[θ_min_]; ${}^{\text{d}}\lambda_{\text{0}}$ represents the crossover wavelength*.

The induced Cotton effects in the Soret region were also examined to investigate the role of TFE on the stabilization of the global structure of the molecule. Figure 3C reports the CD spectra in the Soret region at 0 and 40% TFE (*v/v*). Two negative Cotton effects centered at 357 nm and 463 nm are observed in both CD spectra. In the absence of TFE, the most intense band at 357 nm appears very broad, while it is better resolved at 40% TFE (*v/v*) concentration. Moreover, the intensity of the Cotton effects for all bands increases upon TFE addition. The overall CD data indicate that both secondary and tertiary structures experience a TFE-dependent stabilization. As early reported for this class of compounds (Vitale et al., 2015; Caserta et al., 2018), the helical folding drives the peptide chains to interact with the porphyrin moiety, with consequent stabilization of the sandwiched structure. According to these data, all further spectroscopic and catalytic investigations were performed in aqueous solutions containing 40% (*v/v*) TFE.

#### Coordination Properties of Mn-MC6^*^a by UV-vis pH Titration

The coordination properties of Mn-MC6^*^a were investigated by a UV-vis pH titration, following the changes in the absorption spectrum over a wide pH range (2.0–11.0). The molar absorptivity at 365 nm (ε_365_) was plotted as a function of [H^+^] and the experimental data points were fitted to an equation describing pH-dependent equilibria involving four species (Equation 8, Supporting Information).

**Figure 4:**
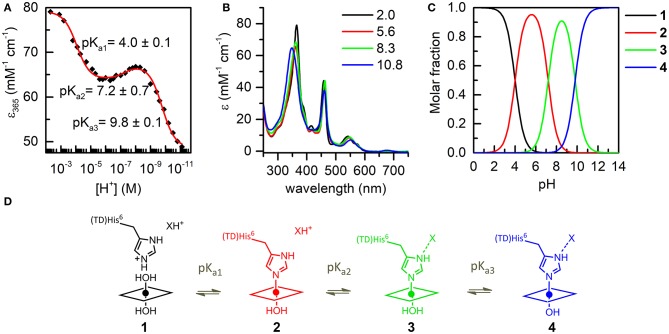
UV-vis pH titration of Mn-MC6^*^a (15 μM) in aqueous solution containing 40% TFE (*v/v*), *T* = 25°C. **(A)** Plot of the ε_365_ (Soret) variation as a function of [H^+^]; data points were fitted according to equation 8 (ESI). **(B)** Absorption spectra of the four species involved in the acid-base equilibria. Curves of different colors represent the different pH values, as indicated in the legend. **(C)** pH-dependent speciation diagram obtained from the titration data. **(D)** Schematic representation of the acid-base equilibria.

The best fit gave three transitions with midpoints at pH 4.0 (pK_a1_), 7.2 (pK_a2_), and 9.8 (pK_a3_) (Figure 4A). Table 2 summarizes the absorption features of the four species participating to the equilibria, whose absorption spectra are reported in Figure 4B. At pH 2, the *bis*-aquo species was predominant (species **1**, Figures 4C and D), characterized by two absorption bands at 365 and 458 nm in the Soret region (see Table 2 and Figure 4B) (Giovannetti et al., 2010). A significant decrease in the absorbance, together with slight wavelength shifts of both bands, occurs as pH increases from 2.0 to 5.4. The component at 365 nm is blue-shifted while the other one is red-shifted. These spectral changes are reasonably attributed to the deprotonation of the His^9^ side-chain (pK_a1_ = 4.0) to give a His-aquo coordination (species **2**) (Low et al., 1998). A further pH increase from 5.4 to 8.4 causes the spectrum to remain substantially unchanged, with a small increase of the Soret extinction coefficient. These spectroscopic features suggest the presence of a deprotonation equilibrium involving a second shell residue (pK_a2_ = 7.2), which does not perturb the first coordination environment and gives rise to species **3**. Upon pH increase from 8.4 to 11.0, substantial spectral changes occur both in the Soret band intensity and position. They may account for the deprotonation of the metal-bound water ligand (pK_a3_ = 9.8), leading to the alkaline form of Mn^III^-MC6^*^a, with the His-hydroxy axial coordination (species **4**).

**Table 2:** UV-vis absorption maxima of Mn-MC6^*^a in H_2_O/TFE solution (60/40 *v/v*) at different pH values.

**pH**	**Species**	**Soret bands λ (nm)**	**Soret ε^a^ (mM^−1^ cm^−1^)**	**Q bands (β,α) λ(nm)**
2.0	1	365, 458	79	542, 573
5.6	2	362, 462	64	547, 575 (sh)
8.3	3	362, 462	68	547, 575 (sh)
10.8	4	348, 460	65	552, 590 (sh)

a *Soret ε refers to the band at lower wavelength*.

These assumptions are further supported by the electrochemical data recorded at the Mn-MC6^*^a complex at different pH values (in the range 5.5–13). The formal potential of the Mn^III^/Mn^II^ determined by cyclic voltammetry was plotted as a function of pH (Figure S2). In the pH range 5.5–10, the formal potential shows no pH-dependence and the E_1/2_ value for Mn^III^/Mn^II^ is −0.31 V *vs*. NHE (Normal Hydrogen Electrode), which is attributed to the His-aquo axial coordination state. The lack of formal potential variation around pH 7 confirms that the acid-base equilibrium occurring around pH ~7 does not alter the metal coordination state. Upon further pH increase to values higher than 10, a decrease of the formal potential was observed, which fully supports the hypothesis that deprotonation of the water ligand occurs, leading to increased stabilization of Mn^III^ vs. Mn^II^ in the His-hydroxy axial coordination state.

It is worth to note here that the E_1/2_ value obtained for the His-aquo coordination state of Mn-MC6^*^a is slightly less negative than those reported for other Mn-porphyrin peptide conjugates with similar coordination states, such as Mn-microperoxidase 8 (Mn-MP8, E_1/2_ = −0.36 V *vs*. NHE at pH 7.5) (Primus et al., 1999) and MnGGH (E_1/2_ = −0.44 V *vs*. NHE) (Ryabova and Nordlander, 2005). This indicates that the relative stability of oxidized vs. reduced state is decreased in the Mn-MC6^*^a complex. In parallel, the water molecule bound at the oxidized Mn^III^ metal ion is more acidic in the Mn-MC6^*^a complex (pK_a3_ = 9.8) as compared with Mn-MP8 (pKa = 11.2) and MnGGH (pKa = 12). A similar effect was already reported in mutants of the H-NOX heme protein (Olea et al., 2010) and attributed to a lower electron density at the metal ion center. This in turn increases its Lewis acidity and thus the Bronsted acidity of the bound water molecule.

As widely reported in the literature (Tezcan et al., 1998; Reedy et al., 2008), heme exposure to solvent also allows to modulate the redox potential. In particular, a hydrophobic core, causing water exclusion from the heme environment, determines an upshift in reduction potential. Thus, the observed shift on the E_1/2_ value in Mn-MC6^*^a, with respect to Mn-MP8 and MnGGH, can be also attributed to a different environment around the His-aquo manganese porphyrin. Both Mn-MP8 and MnGGH lack a distal peptide chain, and therefore one side of the manganese porphyrin is fully exposed to the solvent. On the opposite, the presence of the distal chain in Mn-MC6^*^a creates a different environment, with a hydrophobic patch formed by the Aib methyl groups (Caserta et al., 2018). In such an environment, the reduced form of the redox center, as well as the His-hydroxy oxidized form, would be stabilized, thus causing a positive shift of the reduction potential and a downshift of the bound water pK_a_ value with respect to Mn-MP8 and MnGGH. Finally, the hydrogen bond network within residues of the designed distal pocket (Figure 1) may also play a role in favoring water deprotonation.

### Catalytic Studies

To ascertain the possible role of Mn-MC6^*^a in oxidation chemistry, the formation of high-valent Mn species was first investigated. Subsequently, the catalytic properties were explored in the sulfoxidation of phenyl thioethers, taken as model reaction, and compared with those of Fe-MC6^*^a.

#### Formation of High-Valent Mn Species

**Figure 5:**
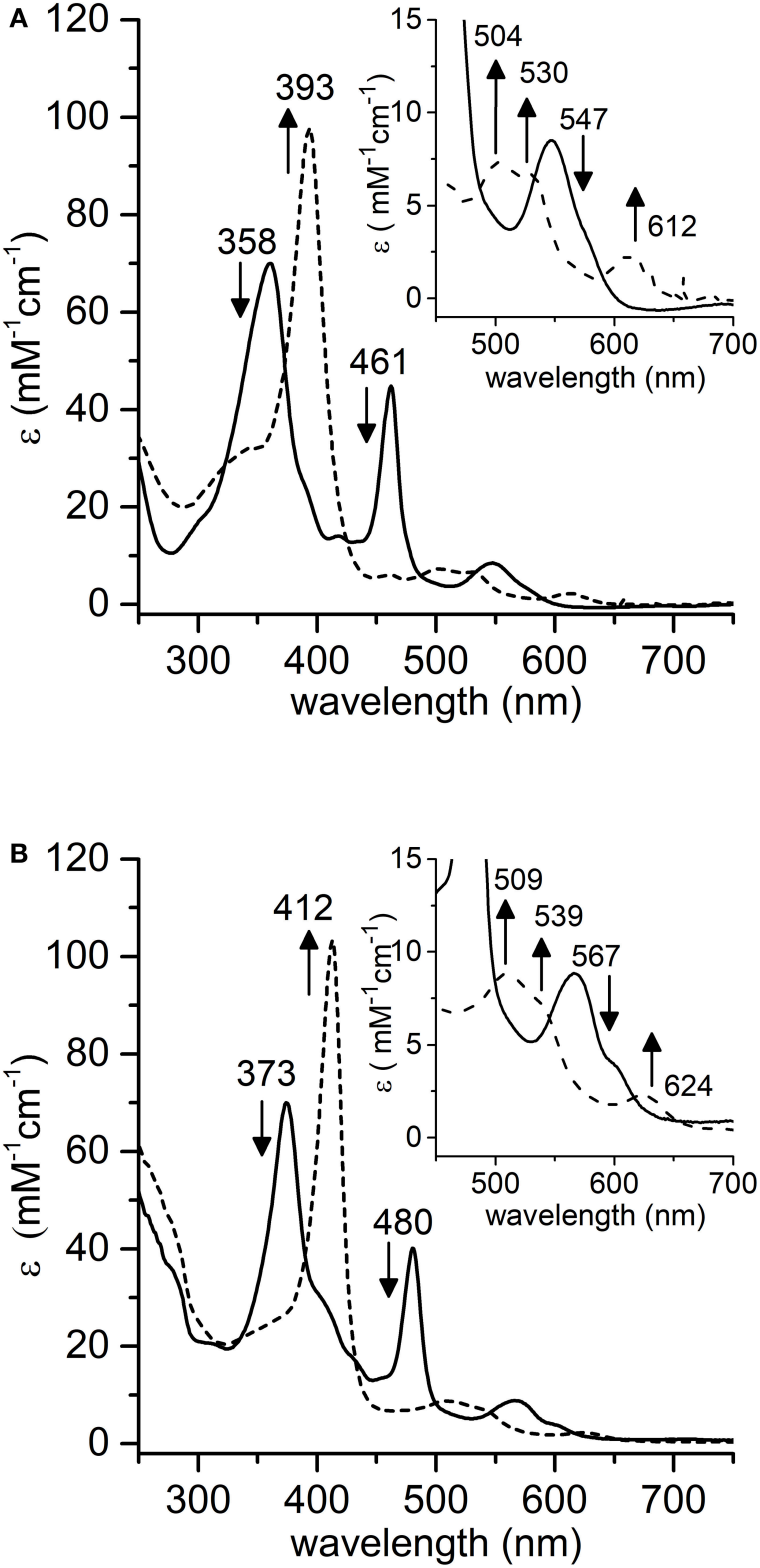
Comparative UV-vis spectra of: **(A)** Mn-MC6^*^a (20 μM, 60 mM carbonate buffer pH 9.5 with 40% *v/v* TFE, T = 25°C) and **(B)** Mn-HRP (8.0 μM, 100 mM phosphate buffer pH 7.0, T = 25°C) before (plain lines) and after (dashed lines) addition of 100 eq. H_2_O_2_. Arrows indicate the direction of absorbance variations.

Spectroscopic studies upon treatment of Mn-MC6^*^a with oxidizing agents were first carried out. Addition of excess hydrogen peroxide (100 eq.) to a buffered solution (60 mM carbonate containing 40% TFE (*v/v*), pH 10) of the complex (20 μM) led to a significant decrease in the intensity of the absorption bands at 358 and 461 nm, with the concurrent formation of a single Soret band at 393 nm. In the visible region, the intensity of the band at 547 nm decreased, while three new bands at 504, 530, and 612 nm appeared (Figure 5A). These spectral changes are consistent with the formation of the oxomanganyl [Mn^IV^=O]^·^^+^ radical cation. Indeed, a very similar absorption spectrum was observed, upon addition of hydrogen peroxide, for Mn-HRP (Figure 5B) (Khan et al., 1996) and Mn-MP8 (Primus et al., 2002) and was identified as the manganese analog of the “Compound I” of heme peroxidases (Dolphin et al., 1971; Hersleth et al., 2006). The observed spectroscopic profile excludes the formation of [Mn^V^ = O] even when stronger oxidizing agents were used (*t*-BuOOH, NaOCl, KHSO_5_) (Figure S3). In order to rule out the possible involvement of hydroxyl radicals derived from photochemical decomposition of hydrogen peroxide (Weiss, 1952), the reaction was also carried out in the presence of d-mannitol (2.0 eq. with respect to H_2_O_2_), acting as radical scavenger (Desesso et al., 1994). The reaction outcome was not altered by the presence of the scavenger (data not shown), thus confirming the reactivity of Mn-MC6^*^a toward hydrogen peroxide. Stability and formation of [Mn^IV^ = O]^·^^+^ depends on the equivalents of added peroxide. Mn^III^-MC6^*^a was quantitatively converted into Compound I upon treatment with 100 eq. H_2_O_2_ and underwent complete bleaching within 20 min (Figure S4A). When lower peroxide concentrations were used, the observed yield of Mn-Compound I formation was lower, but it spontaneously returned to the resting state. Based on the Soret absorbance, the treatment of Mn^III^-MC6^*^a with 10 eq. of H_2_O_2_ led to Mn-Compound I with a 76% yield, while catalyst restoring was around 55% (Figure S4B). Conversely, the reaction with an equimolar amount of H_2_O_2_ provided Mn-Compound I in 45%, and the restoring was almost quantitative (>90%) (Figure S4C). All these data demonstrate that MC6^*^a scaffold is able to host a [Mn^IV^ = O]^·^^+^ species. This behavior distinguishes MC6^*^a from small-molecule Mn-porphyrins, which are typically able to provide both Mn^IV^- and Mn^V^-oxo intermediates (Huang and Groves, 2017). Notably, MC6^*^a is similar to manganese-reconstituted HRP in selectively forming the [Mn^IV^ = O]^·^^+^ species, independently from the nature of the oxidizing agent (Khan et al., 1996, 1998; Nick et al., 2002). However, [HRP-Mn^IV^ = O]^·^^+^ was characterized by a higher stability than [MC6^*^a-Mn^IV^ = O]^·^^+^. Indeed, regardless of the equivalents of H_2_O_2_ added, [HRP-Mn^IV^ = O]^·^^+^ is stable over hours, likely as the result of the the wide delocalization of the radical beyond the porphyrin ring (Khan et al., 1996).

**Figure 6:**
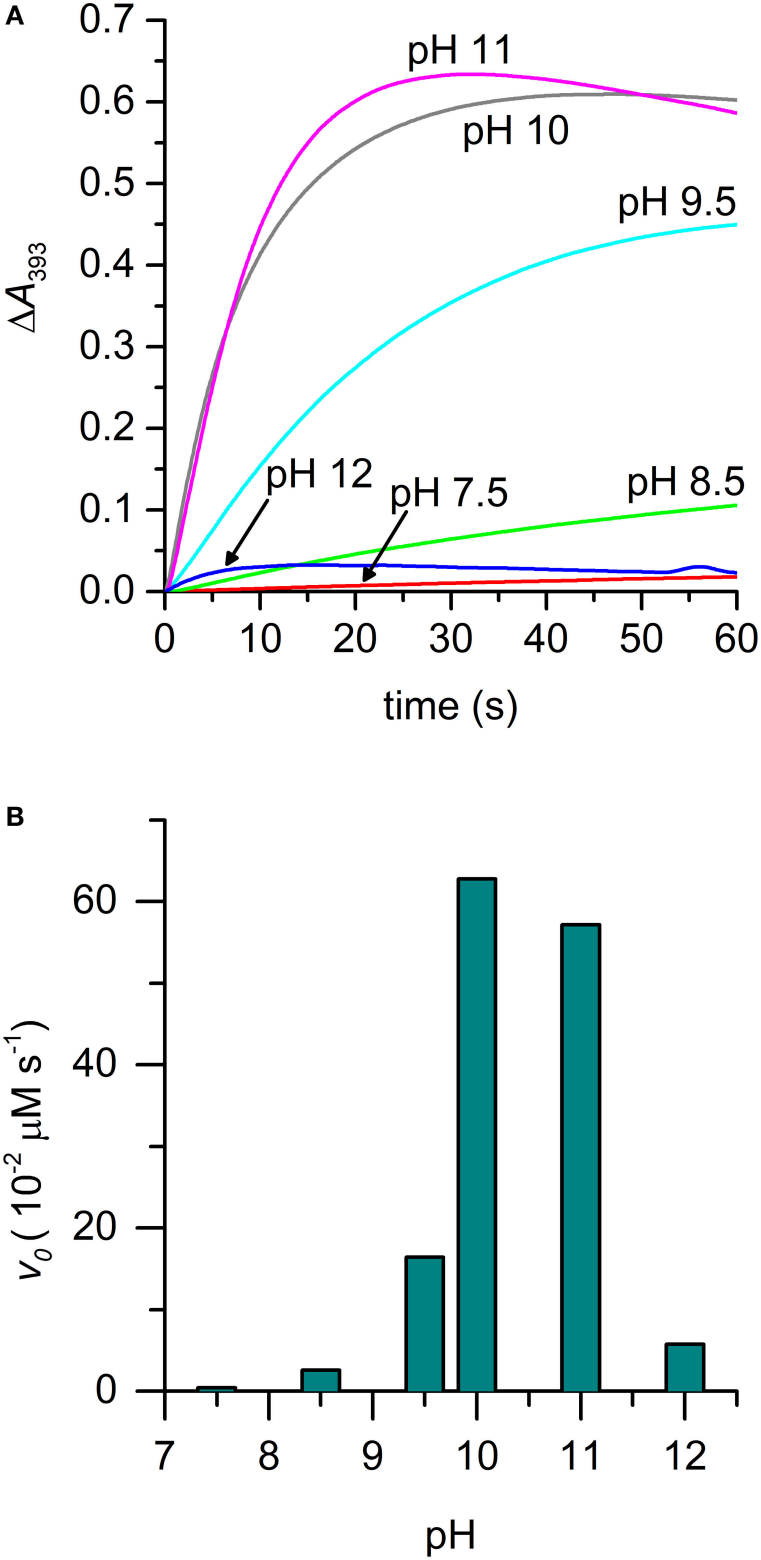
Time-course of [Mn^IV^ = O]^·+^ formation in different pH conditions. **(A)** Experimental curves at different pHs are reported with different colors: pH 7.5, red; pH 8.5, green; pH 9.5, cyan; pH 10, gray; pH 11, magenta; pH 12, blue. **(B)** Plot of the initial rate (*v*_0_) as a function of pH. *Reaction conditions*: Mn-MC6^*^a, 20 μM; H_2_O_2_, 20 μM; T = 25°C. Various buffers were used depending on the pH, as described in the experimental section.

The effect of pH on the rate of [MC6^*^a-Mn^IV^ = O]^·^^+^ formation was also evaluated. The pH-dependent time-course of the reaction was monitored by following the variations in the absorbance at 393 nm upon addition of H_2_O_2_ (1.0 eq.) under different pH conditions (Figure 6A). The formation of Mn-Compound I was remarkably slow at pH values below 7.5 and a negligible amount of the high-valent species was observed. The initial reaction rate was significantly influenced by pH (Figure 6B), reaching the highest value at pH 10 (*v*_0_ = 62.8 10^−2^ μM s^−1^). At pH 11, a similar oxidation rate was observed (*v*_0_ = 57.2·10^−2^ μM s^−1^), even though a subsequent decay of the [Mn^IV^ = O]^·^^+^ species occurred. A conspicuous drop in reactivity was then found above pH 11 (pH 12, *v*_0_ = 5.7·10^−2^ μM s^−1^), which is attributed to decomposition of hydrogen peroxide in the alkaline medium and/or to Compound I instability (Ryabova and Nordlander, 2005). A similar behavior in Mn-Compound I formation was observed when a 10 eq. excess of H_2_O_2_ was used (data not shown).

The alkaline pH value corresponding to the maximum reaction rate in Mn-Compound I formation is not unexpected. Indeed, as previously reported for manganese reconstituted heme-proteins and for model systems, manganese is less efficient than iron in lowering the pK_a_ value of H_2_O_2_. Thus, pH increase is required in order to assist H_2_O_2_ deprotonation, which is necessary for peroxide heterolytic cleavage and Compound I formation (Khan et al., 1996; Primus et al., 2002; Yeh et al., 2002; Cai et al., 2013; Chino et al., 2018). Among Mn^III^ complexes, we evidenced that the pK_a_ of the distal axial ligand is downshifted as compared with the Mn-MP8 complex. This most likely explains that the maximum reactivity for Mn-MC6^*^a is observed at pH 10, two pH units below the value observed for Mn-MP8 (pH 11.9) (Yeh et al., 2002). Based on these pH values, it appears that Mn-MC6^*^a approaches the properties of Mn-HRP (maximum reactivity at physiological pH values) (Khan et al., 1996) better than Mn-MP8.

#### Sulfoxidation of Phenyl Thioethers

**Figure 7:**
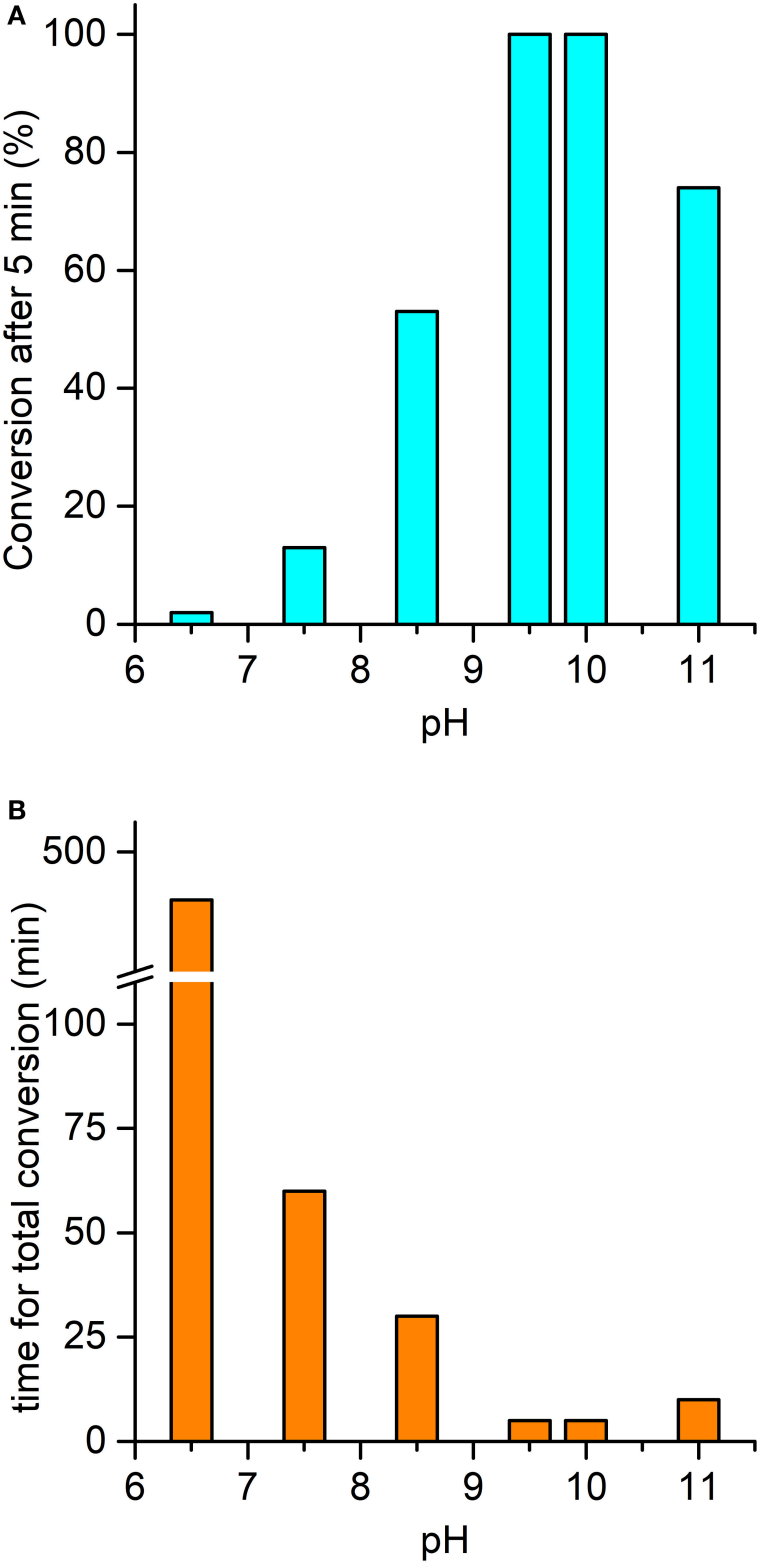
pH-dependent oxidation of thioanisole catalyzed by Mn-MC6^*^a. **(A)** Substrate conversion observed after 5 min of reaction progress; **(B)** time required for a complete conversion of the substrate. *Reaction conditions*: Mn-MC6^*^a, 20 μM; H_2_O_2_, 2.0 mM; thioanisole 2.0 mM; different buffers were used depending on the pH value, as described in the experimental section. At pH 11, a second addition of H_2_O_2_ (after the first 5 min of reaction time) was needed to reach complete conversion (see text). Substrate consumption was monitored by GC-MS analysis of the reaction mixture, using anisole as internal standard.

The ability of the high-valent [Mn^IV^ = O]^·^^+^ species in catalyzing the oxy-functionalization of substrates was investigated. To this aim, the H_2_O_2_-mediated sulfoxidation of phenyl thioethers was chosen as model reaction and followed by GC-MS analysis (Table 3). First experiments were performed with thioanisole (100 eq.) as substrate, under the best conditions for Compound I formation (Mn-MC6^*^a:H_2_O_2_ = 1:100, pH 10.0, 40% *v/v* TFE). Complete conversion of the sulfide into the corresponding sulfoxide was achieved within 5 min by the addition of H_2_O_2_ (Table 3, entry 1). As formation of the high-valent [Mn^IV^ = O]^·^^+^ was found to be strongly pH-dependent, the effect of pH on thioanisole sulfoxidation was also evaluated. Figure 7A reports the substrate conversion at various pHs, by monitoring the consumption of the substrate after 5 min of the reaction progress. A considerable increase in substrate conversion (up to 50-fold) was observed by raising the pH from 6.5 (2% conversion) to 10 (100% conversion). A further pH increase caused a small drop in the conversion (pH 11). Increasing the reaction time, almost complete substrate conversion was observed at any pH value (Figure 7B), although 7 h were required at pH 6.5. As unique exception, at pH 11 the reaction stopped at 74% yield, even after prolonged reaction times. However, further addition of peroxide (100 eq.) led to the complete conversion of the substrate within 5 min. This behavior excludes the lowering of the reaction yield by catalyst inactivation, while suggesting the occurrence of an alternative pathway, currently under investigation. Under optimized reaction conditions (Mn-MC6^*^a, 20 μM; thioanisole, 20 mM; H_2_O_2_, 20 mM, pH 10, 40% *v/v* TFE), the catalyst was able to perform 870 TONs in thioanisole oxidation.

**Table 3:** Enzyme-catalyzed H_2_O_2_-dependent oxidation of thioanisole.

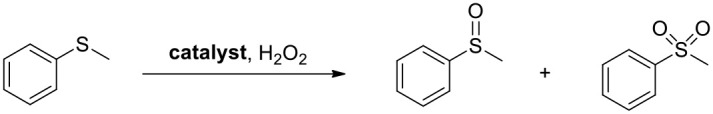					
**Entry**	**Catalyst**	**Catalyst:substrate:H**_2_**O**_2_ **(catalyst concentration)**	**Yield, % (time, min)**	**TON**	**Reference**
1	Mn-MC6^*^a	1:100:100	100 (5)	870^a^	This work
		(20 μM)		
2	Fe-MC6^*^a	1:100:100	97 (5)	1500^b^	This work
		(20 μM)		
3	Mn-HRP	1:100:100	4 (5)	4	This work
		(9 μM)		
4	Fe-HRP	1:30:40	95 (60)	28	Colonna et al., 1992
		(330 μM)		
5	Cr-salophen-Mb(H64D/A71G)	1:100:100	NA^c^	NA^c^	Ohashi et al., 2003
		(10 μM)		
6	Mn-Cor-BSA	1:50:75	83 (90)	150	Mahammed and Gross, 2005
		(200 μM)		
7	Fe(T*p*CPP)-Xln10A	1:425:175	85 (138)	145	Ricoux et al., 2009
		(20 μM)		
8	Mn-salen-Mb(T39C/L72C)	1:40:40	17 (10)	7^d^	Garner et al., 2011
		(130 μM)		
9	Fe-T*p*SPP-NCS-3.24	1:500:500	1.3 (120)	6.5^d^	Sansiaume-Dagousset et al., 2014
		(5 μM)		
10	CoL-BSA	1:100:150	98 (1,680)	98^d^	Tang et al., 2016
		(2.7 μM)		

a *TON was determined using a 1:1000:1000 catalyst:substrate:H_2_O_2_ ratio*.

b *TON was determined using a 1:2000:2000 catalyst:substrate:H_2_O_2_ ratio*.

c *Yield and TON not available from the reference. The reported reaction rate is 78 10^-3^ TON min^-1^*.

d *TON was calculated based on the reported yield and catalyst:substrate ratio*.

Mn-MC6^*^a was also screened in the sulfoxidation of several phenyl sulfides, such as *p*-chlorothioanisole, *p*-nitrothioanisole, *p*-methoxythioanisole, cyclopropyl-phenyl sulfide. A similar reactivity was observed, regardless the presence of activating/deactivating groups by electronic or steric effects (Table 4). These results demonstrate that Mn-MC6^*^a is able to convert phenyl thioethers into the corresponding sulfoxides with high yields. The reactions were found to proceed with chemoselectivity between sulfoxide and sulfone products, as no traces of over-oxidized products (e.g., aryl sulfones) were detected. The only exception was found with *p-*chlorothioanisole, in which 13% of sulfone formation was observed (Figures S5–S9). Unfortunately, no detectable enantiomeric excess was observed.

**Table 4:** Mn-MC6^*^a-catalyzed oxidation of thioethers.

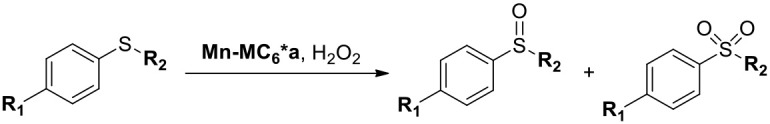			
**Entry**	**R**_1_	**R**_2_	**Conversion (%)**^a^
1	H	Me	100
3	Cl	Me	87^b^
4	NO_2_	Me	100
5	MeO	Me	100
6	H	C_3_H_5_	100

Reaction conditions: Mn-MC6^*^a, 20 μM; sulfide, 2 mM; H_2_O_2_, 2 mM (1:100:100 catalyst:substrate:oxidant ratio), 60 mM carbonate buffer with 40% TFE (v/v). No traces of the corresponding sulfone were detected by GC-MS analysis, unless otherwise specified.

a *Substrate consumption after 5 min of reaction progress*.

b *A small amount (13%) of sulfone was detected*.

**Figure 8:**
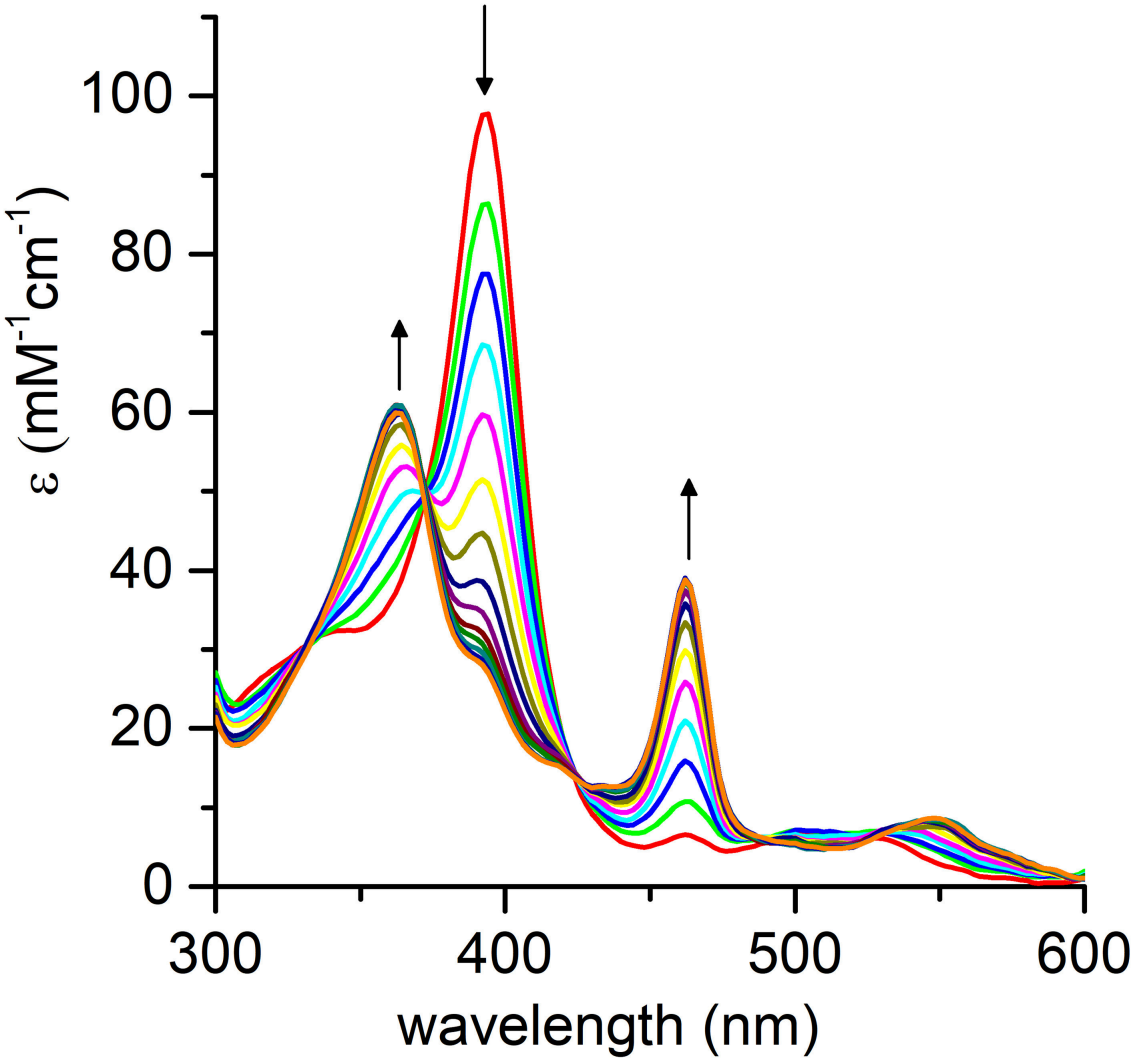
Evolution of the UV-vis absorption spectrum of [Mn^IV^ = O]^·^^+^ upon addition of thioanisole (100 eq.); arrows indicate the direction of absorbance variations. *Reaction conditions*: Mn-MC6^*^a, 20 μM; H_2_O_2_, 2.0 mM; thioanisole 2.0 mM; 60 mM carbonate buffer with 40% TFE (*v/v*), pH 9.5; T = 25°C.

The pH-dependent profile of thioanisole sulfoxidation (Figure 7A) well correlates with the pH-dependent formation of the high-valent [Mn^IV^ = O]^·^^+^ species (Figure 6B). This finding strongly suggests a direct involvement of the Mn-oxo species in substrates oxy-functionalization. To shed light on the reaction mechanism, Mn-MC6^*^a catalyzed thioanisole oxidation was performed with ^18^O-labeled hydrogen peroxide as the oxidant. GC-MS analysis revealed that the reaction with H${}_{2}^{18}$O_2_ produced sulfoxide with 96% ^18^O-labeled oxygen (Figure S6). This result indicates a peroxygenase-like mechanism, with the oxygen incorporated into the sulfoxide deriving from H${}_{2}^{18}$O_2_ through an oxygen transfer mechanism from the Mn-oxo intermediate. The Mn-MC6^*^a-catalyzed oxidation of thioanisole was also monitored by UV-vis spectroscopy (Figure 8), in order to elucidate whether the reaction mechanism occurs *via* a direct oxygen transfer, or by a two-steps, single-electron transfer process, similarly to HRP (Goto et al., 1999). Addition of H_2_O_2_ (2 mM) to a buffered solution (pH 9.5) of the catalyst (20 μM) led to the formation of Compound I. Immediate addition of thioanisole (2 mM) to the reaction mixture led to the rapid disappearance of the bands related to Mn-Compound I and the concurrent return to the catalyst resting state. The presence of isosbestic points at 332, 373, 424, and 491 nm suggests a single-step conversion from [Mn^IV^ = *O*]^·+^ to Mn^III^. This observation indirectly excludes a reaction mechanism involving successive one-electron transfers. Conversely, these data are consistent with a direct oxygen transfer (Goto et al., 1999), involving the nucleophilic attack of the sulfide to Compound I. Further, the almost complete recovery of Mn^III^-MC6^*^a (87%) revealed negligible catalyst degradation.

In order to evaluate the effect of metal ion in MC6^*^a peroxygenase activity, Fe^III^-MC6^*^a was also screened toward thioanisole sulfoxidation. Under optimal conditions for Compound I formation (pH 6.5, 50% *v/v* TFE) (Caserta et al., 2018), Fe-MC6^*^a catalyzed the almost complete conversion of the substrate (97% conversion after 5 min by the addition of H_2_O_2_, Table 3 entry 2) to the corresponding sulfoxide. A drop in the reactivity was observed at pH 9.5 (62% conversion after 5 min by the addition of H_2_O_2_). Similarly to Mn-MC6^*^a, no traces of over-oxidized products (e.g., aryl sulfones) were detected. Further, experiments with ^18^O-labeled hydrogen peroxide indicated the formation of the sulfoxide with 94% ^18^O-labeled oxygen (data not shown). All these results strongly suggest catalysis by Fe^III^-MC6^*^a through a peroxygenase-like mechanism, similarly to Mn^III^-MC6^*^a. Under optimized reaction conditions (Fe-MC6^*^a, 20 μM; thioanisole, 100 mM; H_2_O_2_, 40 mM, pH 6.5, 50% *v/v* TFE), the catalyst was able to perform 1,500 TONs in thioanisole oxidation.

Reactivity toward thioanisole sulfoxidation as a function of pH clearly indicated different behaviors among the iron and manganese complexes. Indeed, complete conversion was observed at pH 9.5 for Mn-MC6^*^a, whereas conversion was lower (62%) at this pH when using Fe-MC6^*^a as catalyst. On the opposite, in the condition of maximum reactivity for the iron complex (pH 6.5, 97% conversion), Mn-MC6^*^a shows negligible activity (2% yield). This finding clearly reflects the different ability of iron and manganese in activating hydrogen peroxide (Chino et al., 2018). Nevertheless, it is important to outline that the MC6^*^a scaffold is able to drive the reactivity of the high-valent metal-oxo species toward peroxygenase catalysis at very different pH values, simply changing the metal ion.

## Conclusion

This work was focused on the spectroscopic and functional characterization of Mn-MC6^*^a, an artificial metalloenzyme belonging to the mimochrome family. All the results demonstrate that MC6^*^a scaffold is able to accommodate manganese and tune its reactivity. In particular, spectroscopic characterization provided evidences for the formation, upon treatment of Mn^III^-MC6^*^a with hydrogen peroxide, of the oxomanganyl [Mn^IV^ = *O*]^·+^ radical cation. Notably, this species is able to catalyze peroxygenase reactions, through a direct oxygen-transfer pathway, with high conversion yields. A similar peroxygenase activity was also detected for Fe^III^-MC6^*^a, previously demonstrated to be one of the most stable and efficient catalysts with peroxidase activity (Caserta et al., 2018).

Comparison of Fe-MC6^*^a and Mn-MC6^*^a with native and manganese-reconstituted HRPs revealed interesting features of our miniaturized protein scaffold. It is well known that HRP is evolved for the reduction of hydroperoxides because of its binding pocket, which is able to properly accommodate H_2_O_2_. Its high-valent iron-oxo intermediate (Compound I) usually catalyzes one– or two–electron oxidations of several substrates with high efficiency (Poulos, 2014). Native HRP has much lower peroxygenase activity, in which the two-electron reduction of Compound I is coupled to the transfer of the ferryl oxygen to a substrate. Only exception is the sulfoxidation of thioanisoles and related sulfur compounds (Colonna et al., 1992). However, mutations at the distal site are needed to enhance HRP peroxygenase activity, thus highlighting that inaccessibility of the ferryl oxygen suppresses direct interaction with substrates (Ozaki and Ortiz de Montellano, 1995; Savenkova et al., 1996, 1998). Further, oxidation of substrates by HRP through oxygen transfer is in competition with enzyme inactivation by spontaneous self-oxidation, at a rate dependent on the concentration of oxidants and nature of the substrate (Colonna et al., 1992; Velde et al., 2001). Indeed, enhancement in the peroxygenase activity of peroxidases has been obtained by keeping the concentration of H_2_O_2_ at a low level (Table 3 entry 4) (Colonna et al., 1992; Velde et al., 2001).

Manganese substitution in HRP causes a further decrease in its peroxygenase activity, because the high chemical stability of Mn-HRP Compound I (Nick et al., 2002) completely hinders its reactivity in oxygen-transfer reactions.

For a straightforward comparison with our systems, in this work we analyzed the reactivity of native HRP and Mn-HRP under our experimental conditions, i.e., catalyst/oxidant/substrate 1:100:100. Under these conditions, native HRP showed poor peroxygenase activity (10% conversion), probably because of catalyst inactivation by excess peroxide. Substitution of iron to manganese caused a further decrease in peroxygenase reactivity (4% conversion yield, Table 3 entry 3). These data underline the power of our miniaturization approach in the construction of artificial metalloenzymes, affording a designed scaffold, MC6^*^a, able to host different metal ions. Indeed, swapping of iron to manganese leaves the catalytic activity almost unchanged. This behavior endows it with broad catalytic activity and versatility, being able to steer the active species toward peroxidative and/or peroxygenative catalysis. The finding that oxy-functionalization of thioanisole can be performed with similar yields at two very different pHs, simply changing iron to manganese (pH 6.5 and 9.5, respectively) can be important for applications with pH- sensitive substrates. Finally, it is worth noting that the activity of Fe– and Mn-MC6^*^a places them among the most active artificial biocatalysts available to date in the H_2_O_2_-mediated thioanisole oxidation (Ohashi et al., 2003; Mahammed and Gross, 2005; Ricoux et al., 2009; Garner et al., 2011; Sansiaume-Dagousset et al., 2014; Tang et al., 2016). In particular, a comparison of the catalytic performance in terms of TON between our catalysts and a variety of artificial metalloenzymes (Table 3) reveals that both Mn-MC6^*^a and Fe-MC6^*^a are robust catalysts, being able to perform 870 and 1,500 TONs, respectively. Further experiments are currently ongoing in order to shed light on the different robustness of the two catalysts.

In conclusion, the results herein reported demonstrate that MC6^*^a scaffold fills the middle-ground between native proteins and small-molecule catalysts. Despite its small structure, it holds enzyme-like structural features by tuning the reactivity of the metal center thanks to a properly designed distal site. It also embodies some typical features of metalloporphyrin catalysts, such as an easily accessible distal site for oxygen-transfer reactions (Neu et al., 2015). Future work will be devoted to exploring the catalytic versatility of Mn-MC6^*^a and Fe-MC6^*^a in promoting different reactions of synthetic and/or biotechnological interest.

## Materials and Methods

Mn^II^ acetate and glacial acetic acid was purchased from Sigma Aldrich. HPLC grade solvents were employed for chromatographic analyses and purifications (Romil). Solvents with higher degree of purity were used in the preparation of solutions for LC-MS, GC-MS, UV-Vis, and CD investigations (Ups grade, Romil). Phosphate and carbonate sodium salts (mono– and dibasic), for buffers preparation, thioanisole (analytical standard), hydrogen peroxide (H_2_O_2_) solution (30% *w/w* in water) and isotope labeled hydrogen peroxide (H${}_{2}^{18}$O_2_) solution (3% *w/w* in water) were supplied by Sigma Aldrich. 4-nitrothioanisole, 4-chlorothioanisole, 4-methoxythioanisole and cyclopropyl-phenyl-sulfide employed in catalytic assays were all provided (98% purity) by Alfa Aesar.

HPLC and LC-MS analysis were performed with a Shimadzu LC-10ADvp equipped with an SPDM10Avp diode-array detector. ESI-MS spectra were recorded on a Shimadzu LC-MS-2010EV system with ESI interface, Q-array-octapole-quadrupole mass analyzer and Shimadzu LC-MS solution Workstation software for data processing. Flash Chromatography was performed using a Biotage Isolera flash purification system, equipped with a diode-array detector.

Atomic absorption measurements were performed using a Shimadzu AA-7000 Series equipped with a graphite furnace atomizer. UV-vis analysis was performed on Cary Varian 50 Probe UV Spectrophotometer equipped with a thermostated cell holder and a magnetic stirrer. CD measurements were carried out on Jasco J-815 dichrograph, equipped with a thermostated cell holder (JASCO, Easton, MD, USA). GC-MS analyses were performed by a Shimadzu GCMS-QP2010 SE system equipped with an EI MS source and a quadrupole array as MS analyzer.

### Synthesis and Purification of Mn-MC6^*^a

*Apo*-MC6^*^a was synthesized combining methods of solution and solid-phase peptide synthesis, as previously described by us (Caserta et al., 2018).

Manganese ion was inserted, according to the acetate method procedure (Dolphin, 1978), slightly modified by us. Mn^II^ acetate (10 eq.) was added to a solution of pure *apo*-MC6^*^a in 2/3 TFE/AcOH (*v/v*) (C${}_{\text{MC}6^{*}a}$ = 2.0·10^−4^ M), and the reaction mixture was kept at 50°C for 24 h, refluxing under nitrogen atmosphere. The reaction was monitored by analytical reverse phase HPLC, using a C18 column (4.6 mm·150 mm; 5 μm), eluted with a linear gradient of acidic acetonitrile (0.1% TFA *v/v*) in acidic water (0.1% TFA *v/v*), from 10 to 50% over 30 min, at 1.0 mL·min^−1^ flow rate.

Once the reaction was completed, the solvent was removed under vacuum and the product was purified from the excess of manganese acetate by Reverse Phase-Flash Chromatography, on a SNAP KP-C18-HS 30 g column, using a gradient of acetonitrile in 0.1% aqueous TFA, 5% to 95% over 2 column volumes, at 25 mL·min^−1^ flow rate.

### Determination of Molar Absorptivity of Mn-MC6^*^a

Molar absorptivity (ε) at 365 nm was determined for Mn-MC6^*^a using Atomic Absorption spectroscopy (AAS) and UV-vis spectroscopy. In detail, a stock solution of the catalyst (≈2.0·10^−4^ M) was prepared in H_2_O 0.1% TFA (*v/v*) and its concentration was determined after mineralization using AAS. Mineralization was carried out by treating aliquots (50 μL) of stock solution with HNO_3_ (200 μL) at 95°C for 2 h. Then, the samples were diluted with H_2_O 2% HNO_3_ (*v/v*) to a final concentration of Mn^III^ ions of ≈2 ppb. Manganese concentration in the stock solution was determined by comparison with a calibration curve obtained using standards. Since metal:catalyst ratio is 1:1, the catalyst concentration was determined. Mn-MC6^*^a stock solution as determined via AAS was used to prepare different diluted samples, which were used to obtain the ε value at 365 nm by UV-Vis spectroscopy. The absorbance at 365 nm was plotted as a function of catalyst concentration (Figure S10). The experimental data were fitted to a Lambert-Beer's law, giving a ε_365_ = (7.86 ± 0.08)·10^4^ M^−1^ cm^−1^.

### CD Experiments

Solutions of Mn-MC6^*^a were prepared at C = 2.0·10^−5^ M in 5 mM phosphate buffer at pH 6.5, using various TFE percentages from 0 to 50% (*v/v*). Far-UV CD spectra were collected from 260 to 190 nm using cells of 0.1 cm path length; spectra in the Soret region were collected from 500 to 300 nm using cells of 1 cm path length. All spectra were recorded at 0.2 nm intervals with a 20 nm min^−1^ scan speed, at 2 nm bandwidth and at 16 s response. All measurements were performed at 25°C.

### pH Titration Experiments

Solutions of Mn-MC6^*^a were prepared at C = 1.5 × 10^−5^ M, in a mixture of H_2_O and TFE (60/40 *v/v*). Solutions of NaOH (1, 0.1, and 0.01 M) and TFA (0.1 and 1% *v/v* in water) were used to adjust the pH of the samples (dilution was < 1% and considered in the final data). The model employed for data fitting is reported in the Supporting Information (eqn. 1–8).

### Electrochemistry Experiments

Cyclic voltammetry was performed in a small volume home-made cell by using an Autolab PGSTAT-12 potentiostat controlled by GPES-4 software. The cell was constituted by a small cylindrical vial surrounded by a septum perforated to allow positioning of the three electrodes as well as the argon tube. The working electrode was a glassy carbon electrode (CHInstruments Inc.), reference electrode was Ag/AgCl (WPI, Dri-ref, + 0.2 V vs. NHE at 25°C) and counter electrode was a platinum wire. The cell was filled with 0.35 mL of a 0.1 mM Mn-MC6^*^a solution, prepared from dilution of a 1 mM stock Mn-MC6^*^a solution in milliQ water (35 μL) into a mixture of the appropriate buffer (175 μL of a 250 mM stock solution) and TFE (140 μL). The working electrode was polished on 1 and 0.1 μm alumina and the solution degassed by argon bubbling prior measuring cyclic voltammograms at 10 mV·s^−1^ at room temperature. In the text, potentials are quoted *vs*. NHE.

### Reaction of Mn^III^-MC6^*^a With H_2_O_2_

Solutions of Mn-MC6^*^a (C = 20 μM) were prepared in 60 mM carbonate buffer containing 40% TFE (*v/v*) at pH 10. Reactions were initialized by addition of different amounts of hydrogen peroxide (1, 10, 100 eq.) from properly diluted stock solutions of H_2_O_2_ in water. Reaction progress was followed by continuously collecting UV-Vis spectra in the 250–750 nm region using a 9,600 nm/min scan speed. Catalyst recovery was estimated based on the Soret absorbance when no more changes were observed. Similar experiments were performed with a prepared sample of Mn-HRP. In these cases, 8.0 μM protein in 100 mM phosphate buffered solutions at pH 7 were employed.

In the pH-dependent experiments, reactions were initiated by addition of 1.0 eq. H_2_O_2_ to a buffered solution of the catalyst (C = 20 μM) containing 40% TFE (*v/v*). Different buffers were used depending on the pH: pH 6.5–8.5, phosphate buffer; pH 9.5–10 carbonate buffer. The pH of the solutions was adjusted with NaOH in the experiments at higher pH values. Reactions were monitored over 60 s by collecting the single-wavelength absorbance traces at 393 nm. The initial rate (*v*_0_) was determined, for each pH value, as the slope of the reaction progress curve at t = 0 s.

All experiments were performed at T = 25°C, under magnetic stirring, using quartz cells of 1 cm path length.

### Preparation of Mn-HRP

Mn-protoporphyrin IX, *apo*-HRP, and Mn-HRP were prepared according to literature procedures (Yonetani and Asakura, 1969). The insertion of Mn porphyrin into *apo*-peroxidase was assessed by UV-vis spectroscopy in comparison with literature data (Yonetani and Asakura, 1969; Khan et al., 1996). The homogeneity of the sample was ascertained by analytical Gel Filtration Chromatography (GFC), using a Yarra SEC-2000 column (7.8 mm·300 mm; 3 μm), with an isocratic flow of 0.05 M sodium phosphate 0.3 M NaCl pH 6.8 as mobile phase, at a flow rate of 0.35 mL min^−1^. Fe-HRP was analyzed under the same experimental conditions for comparison (Figure S11). Molecular weight of the samples was determined based on a calibration curve obtained with standards (Figure S12). The GE Healthcare LMW Calibration kit, containing Conalbumin, Ovalbumin, Carbonic anhydrase and Ribonuclease A, all at a concentration of 1 mg/mL, was used for calibration. To prepare the calibration curve, K_av_ of the proteins were calculated as follows:

$$\begin{array}{l} K_{av}=\;\frac{V_{e}-V_{0}}{V_{c}-V_{0}} \end{array}$$

where V_0_ is column void volume, V_c_ is the geometric volume of column, V_e_ is the elution volume of the protein. A value of 48 kDa was found for the molecular weight of both Fe-HRP and Mn-HRP.

### General Procedure for Sulfoxidation Reactions

Stock solutions of substrates (thioanisole, TA; *p-*chlorothioanisole, *p*CTA*; p*-nitrothioanisole, *p*NTA; *p*-methoxythioanisole, *p*MTA; cyclopropyl-phenyl sulfide, CPPS) were prepared dissolving a known amount of neat sulfide in TFE to a final concentration of 0.1 M.

All reactions were carried out at 20 μM catalyst concentration in 60 mM buffered solution with 40% TFE (*v/v*). Reactions with Mn-HRP and Fe-MC6^*^a as catalysts were performed in absence and in presence of 50% (*v/v*) TFE, respectively. Depending on the pH value, different buffers were used: pH 6.5–8.5, phosphate buffer; pH 9.5–10.0, carbonate buffer. Reaction at pH 11 was performed in a 60 mM carbonate solution, whose pH was adjusted with NaOH. All reactions were carried out at room temperature and under magnetic stirring. All assays were performed at 1:100:100 catalyst:substrate:oxidant ratio. TON was determined using a 1:1,000:1,000 ratio and a 1:2,000:2,000 ratio for Mn- and Fe-MC6^*^a, respectively.

The catalyst was preloaded with substrate prior to addition of hydrogen peroxide. Reaction was then initialized by addition of hydrogen peroxide from a solution of 0.1 M H_2_O_2_ in water. For TA, reaction was carried out also using ^18^O-labeled hydrogen peroxide to test the peroxygenase activity of Fe- and Mn-MC6^*^a. A solution of 90% ^18^O-enriched H_2_O_2_, at 0.1 M concentration was used. Reaction progress was monitored by GC-MS, using anisole as internal standard. At different times, an aliquot of the reaction mixture (50 μL) was diluted with an equal volume of H_2_O 0.1% TFA (*v/v*) and extracted with ethyl acetate (100 μL). Residual water was removed from the organic phase with anhydrous sodium sulfate. GC-MS analysis of the organic phase was performed using a Rxi-5Sil-MS Column with helium as carrier gas. A linear gradient from 80°C to 230°C with a rate of 18°C min^−1^ was used for thioanisole and *p*CTA; a linear gradient from 70° to 200°C with a rate of 15°C min^−1^ was used for CPPS; a linear gradient from 70° to 200°C with a rate of 18°C min^−1^, and then from 200°C to 300°C with a rate of 40°C min^−1^ was used for *p*NTA and *p*MTA. MS analysis was performed in TIC (Total Ion Current) mode, exploring a range of m/z from 50 to 250 Th. The grade of conversion at different reaction times was determined based on substrate consumption, using the following equation:

$$Conversion\;\left(\%\right)=\;\frac{{\left(\frac{A_{sub}}{A_{I.Std}}\right)}_{0}-{\left(\frac{A_{sub}}{A_{I.Std.}}\right)}_{x}}{{\left(\frac{A_{sub}}{A_{I.Std.}}\right)}_{0}}\;\cdot 100$$

where A_sub_ and A_I.Std._ are the peak areas of substrate and internal standard, respectively, in the GC-MS TIC chromatogram. The subscript *0* indicates the trace acquired prior to addition of peroxide, while the subscript *x* is a specific time during the reaction course.

Control reactions in the absence of catalyst were also performed and gave no reaction progress.

## Author Contributions

LL and DD performed the spectroscopic and catalytic experiments. GZ synthesized and purified the molecule; VB performed the electrochemical measurements. MC, FN, and OM contributed to the design and discussion of the experiments. LL organized all the information in both main text and Supplementary Material. VP and AL analyzed results and provided critical feedback. AL and FN wrote the manuscript in consultation with the remaining authors.

### Conflict of Interest Statement

The authors declare that the research was conducted in the absence of any commercial or financial relationships that could be construed as a potential conflict of interest.
