# Supplementary material for: Mn-Mimochrome VI*a: An Artificial Metalloenzyme With Peroxygenase Activity
Source: Front Chem. 2018 Dec 4;6:590. doi: 10.3389/fchem.2018.00590 (PMC6288486; doi:10.3389/fchem.2018.00590)
Supplement: Supplementary file 1 [file Data_Sheet_1.PDF]

Supplementary Material

Mn-Mimochrome VI*a: an Artificial Metalloenzyme with Peroxygenase Activity

Linda Leone, Daniele D'Alonzo, Véronique Balland, Gerardo Zambrano, Marco Chino, Flavia Nastri, Ornella Maglio, Vincenzo Pavone and Angela Lombardi*

*** Correspondence:** prof. Angela Lombardi: alombard@unina.it

1 Supplementary Data

pH titration data fitting

The pH-dependent equilibrium is described by the following equation:

$$AH_3^{3+} \xrightarrow{Ka1} BH_2^{2+} + H^+ \xrightarrow{Ka2} CH^+ + H^+ \xrightarrow{Ka3} D + H^+ (Eq. 1)$$

The equilibrium constants, Ka_1, Ka_2 and Ka_3, can be expressed as:

$$K_{a1} = \frac{[BH_2^{2+}] \cdot [H^+]}{[AH_3^{3+}]} \; ; \; K_{a2} = \frac{[CH^+] \cdot [H^+]}{[BH_2^{2+}]} \; ; \; K_{a3} = \frac{[D] \cdot [H^+]}{[CH^+]} \quad (Eq. 2)$$

The total concentration (C_{tot}) of Mn^{III}-MC6a can be expressed as sum of the four species:

$$C_{tot} = [AH_3^{3+}] + [BH_2^{2+}] + [CH^+] + [D] \quad (Eq. 3)$$

The total absorbance (A_{tot}) can be expressed as the sum of the contribution of all the four species:

$$A_{tot} = \varepsilon_A [AH_3^{3+}] + \varepsilon_B [BH_2^{2+}] + \varepsilon_C [CH^+] + \varepsilon_D [D] \quad (Eq. 4)$$

The total concentration and the total absorbance can be indicated in dependence of [D]:

$$C_{tot} = [D] + \frac{[D] \cdot [H^+]}{K_{a3}} + \frac{[D] \cdot [H^+]^2}{K_{a3} \cdot K_{a2}} + \frac{[D] \cdot [H^+]^3}{K_{a3} \cdot K_{a2} \cdot K_{a1}} \quad (Eq. 5)$$

$$A_{tot} = \varepsilon_D \cdot [D] + \varepsilon_C \cdot \frac{[D] \cdot [H^+]}{K_{a3}} + \varepsilon_B \cdot \frac{[D] \cdot [H^+]^2}{K_{a3} \cdot K_{a2}} + \varepsilon_A \cdot \frac{[D] \cdot [H^+]^3}{K_{a3} \cdot K_{a2} \cdot K_{a1}} \quad (Eq. 6)$$

Equations 5 and 6 can be rearranged, giving:

$$A_{tot} = \frac{C_{tot} \cdot \left(\varepsilon_D + \varepsilon_C \cdot \frac{[H^+]}{K_{a3}} + \varepsilon_B \cdot \frac{[H^+]^2}{K_{a3} \cdot K_{a2}} + \varepsilon_A \cdot \frac{[H^+]^3}{K_{a3} \cdot K_{a2} \cdot K_{a1}}\right)}{\left(1 + \frac{[H^+]}{K_{a3}} + \frac{[H^+]^2}{K_{a3} \cdot K_{a2}} + \frac{[H^+]^3}{K_{a3} \cdot K_{a2} \cdot K_{a1}}\right)} \quad (Eq. 7)$$

Equation 7 can be written as:

$$A_{tot} = \frac{\left(A_D + A_C \cdot \frac{[H^+]}{K_{a3}} + A_B \cdot \frac{[H^+]^2}{K_{a3} \cdot K_{a2}} + A_A \cdot \frac{[H^+]^3}{K_{a3} \cdot K_{a2} \cdot K_{a1}}\right)}{\left(1 + \frac{[H^+]}{K_{a3}} + \frac{[H^+]^2}{K_{a3} \cdot K_{a2}} + \frac{[H^+]^3}{K_{a3} \cdot K_{a2} \cdot K_{a1}}\right)} \quad (Eq. 8)$$

A_A, A_B, A_C, A_D, parameters represent the absorbance of the four differently protonated species. Species A-D are referred as **1-4** in the main text. Equation 8 was used to fit the experimental A_{365} (Soret) data points (Figure 4A, main text), which were reported as molar extinction coefficient at 365 nm (ε_{365}).

2 Supplementary Figures and Tables

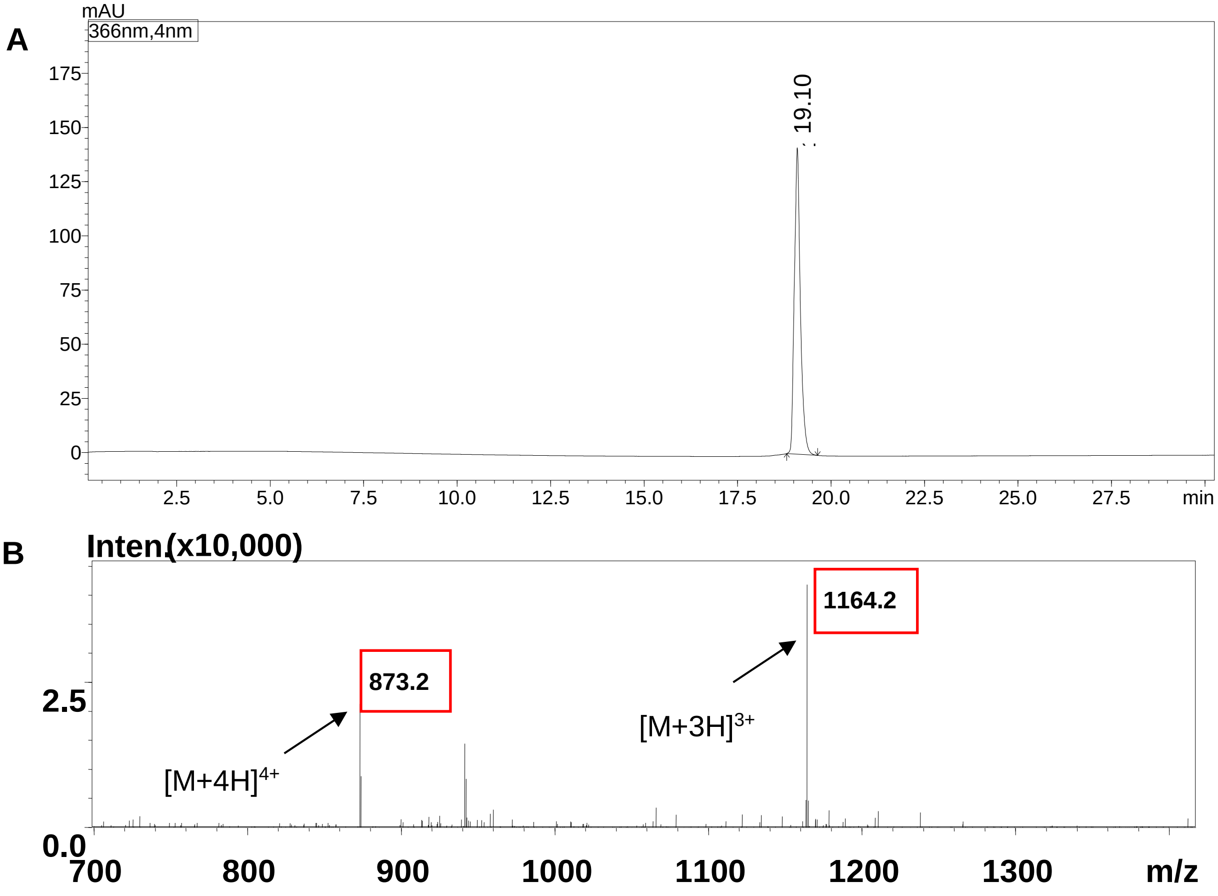

Figure S1: LC-MS chromatogram of pure Mn^{III}-MC6a (A), and its positive ESI-MS m/z spectrum (B). The peaks corresponding to the intact compound, carrying the indicated number of charges (protons), are evidenced. Experimental mass: (3489.2 ± 0.4) Da; Theoretical mass: 3488.8 Da.

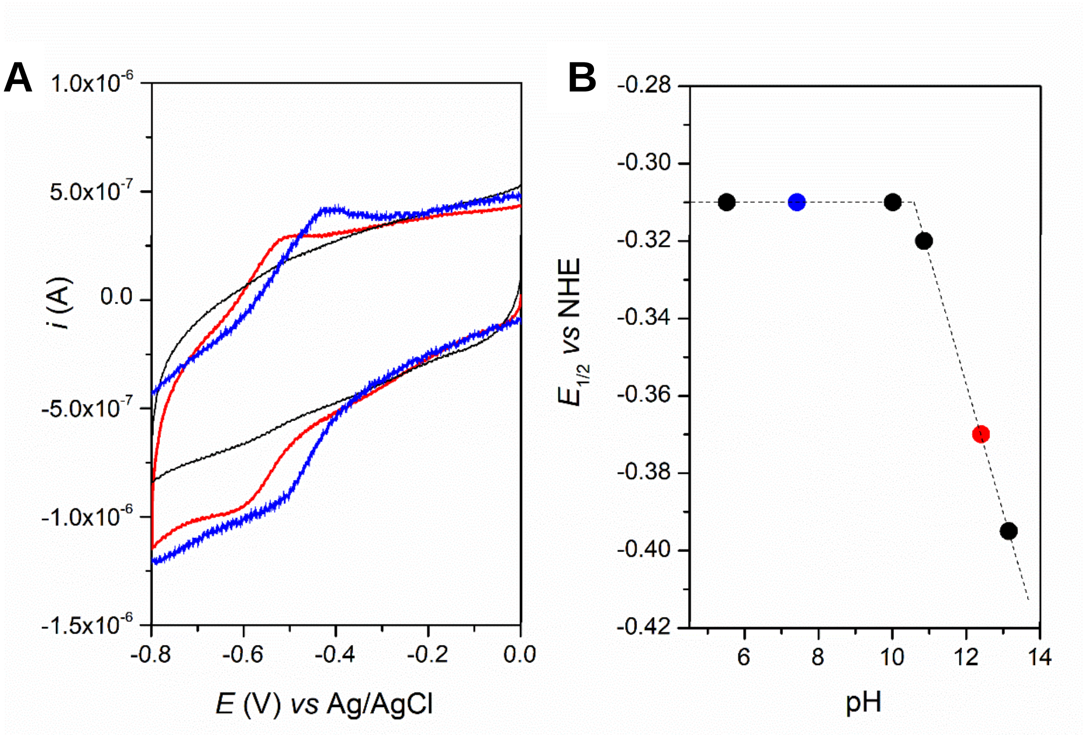

Figure S2: A) Cyclic voltammograms (CVs) of Mn-MC6*a (C = 100 μM in buffer solution containing 30% TFE (*v/v*), T = 25 °C) recorded at 10 $mV \cdot s^{-1}$ scan speed at pH 7.4 (blue trace) and 12.4 (red trace). CV acquired from a solution at pH 12.4 in absence of Mn-MC6*a is also shown (black trace). B) pH-dependence of Mn^{III}/Mn^{II} formal reduction potential ($E_{1/2}$).

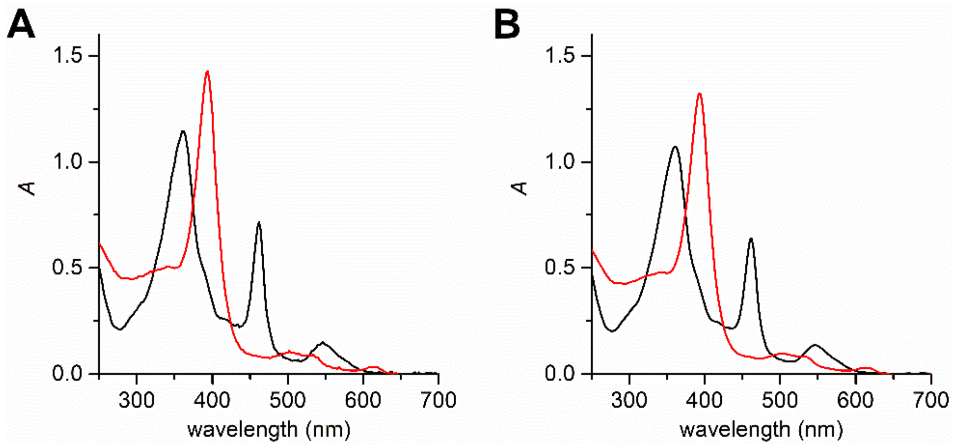

Figure S3: UV-Vis spectra of Mn-MC6*a (C = 20 μM in 60 mM carbonate buffer containing 40% TFE (*v/v*), pH 9.5; T = 25 °C) before (black lines) and after (red lines) treatment with different oxidizing agents: A) NaOCl (1000 eq.); B) $KHSO_5$ (100 eq.). No reaction was observed upon treatment with *t*-BuOOH (100 eq.).

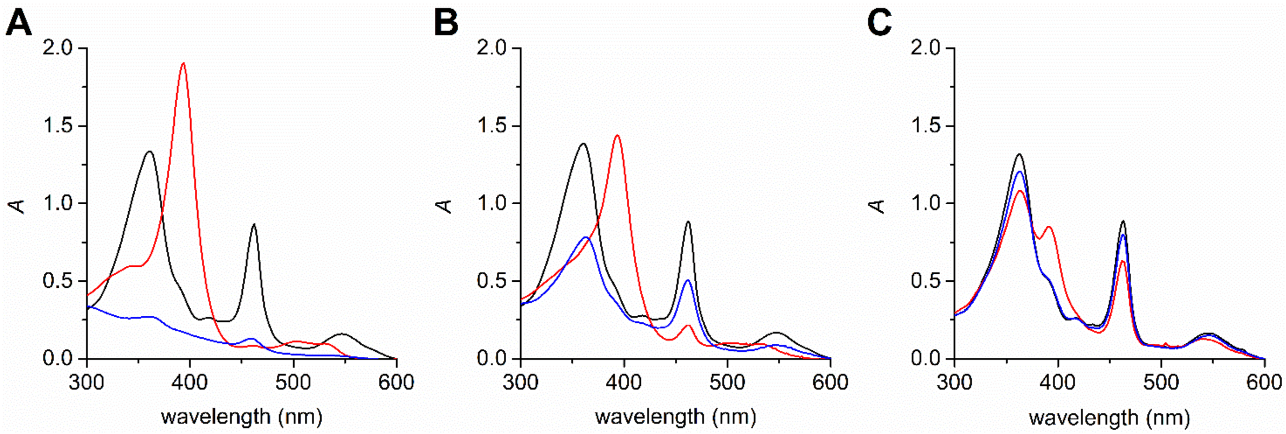

Figure S4: UV-Vis spectra of Mn-MC6*a upon treatment with different amounts of H_2O_2: A) 100 eq.; B) 10 eq. and C) 1 eq. Spectra acquired prior to addition of peroxide are depicted as black lines. Red lines represent the maximal conversion of Mn^{III}-MC6*a into Compound I (observed at t = 5 s, 40 s and 90 s after the addition of peroxide for 100 eq., 10 eq. and 1 eq., respectively). Blue lines represent the spectra acquired after 20 minutes from the addition of peroxide (C = 20 μM in 60 mM carbonate buffer containing 40% TFE (*v/v*), pH 10; T = 25 °C).

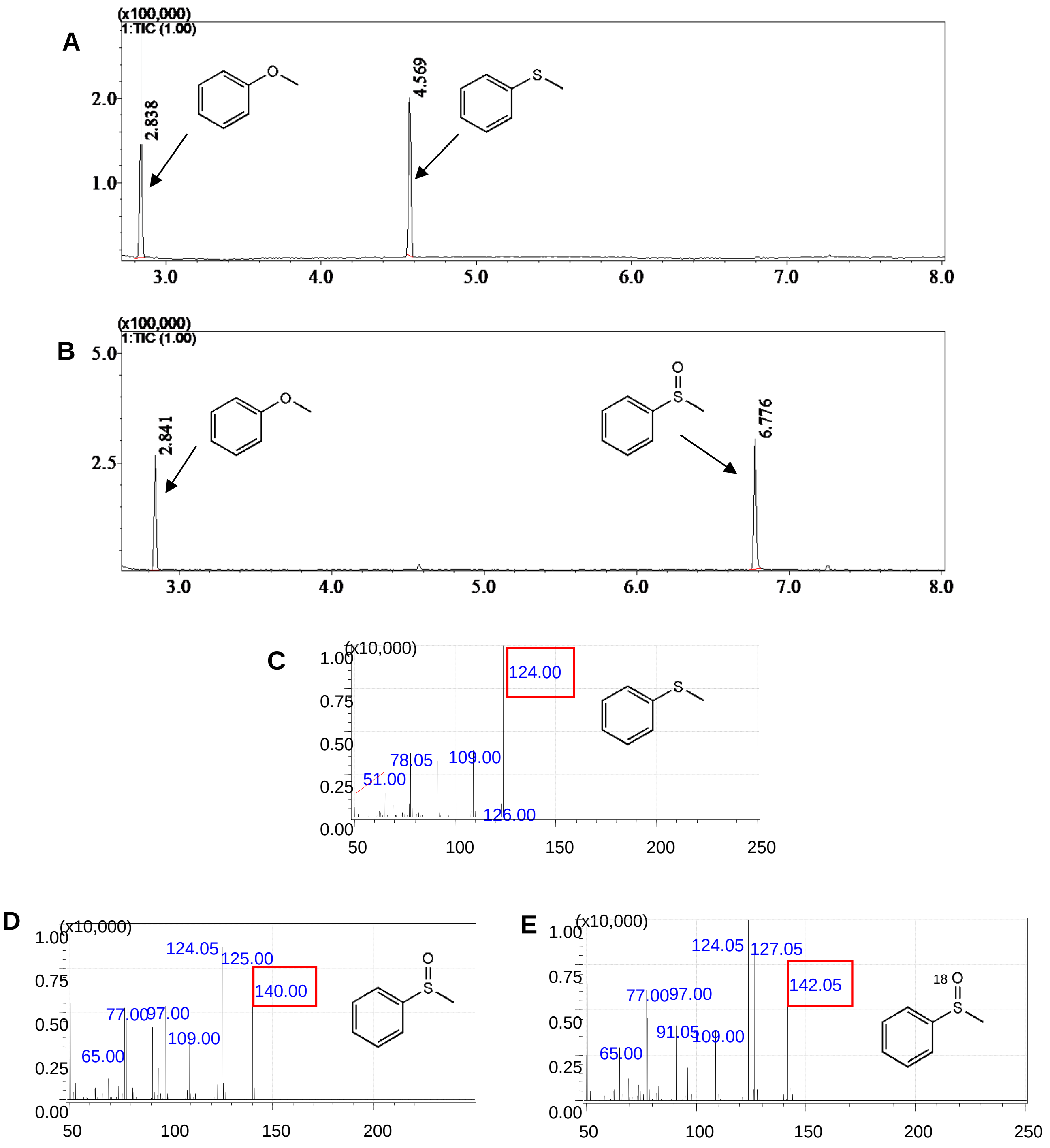

Figure S5: GC-MS TIC chromatogram of thioanisole sulfoxidation: A) before addition of peroxide and B) after reaction completion. Peak eluted at Rt= 2.84 min is relative to anisole (internal standard). C) and D) are the EI-MS spectra of products eluted at Rt = 4.57 min and 6.78 min, which were identified respectively as thioanisole and methyl-phenyl sulfoxide. E) is the EI-MS spectrum of ^{18}O containing methyl-phenyl sulfoxide, obtained when reaction was carried out with $H_2{}^{18}O_2$ as oxidant. Molecular ions are labeled in red.

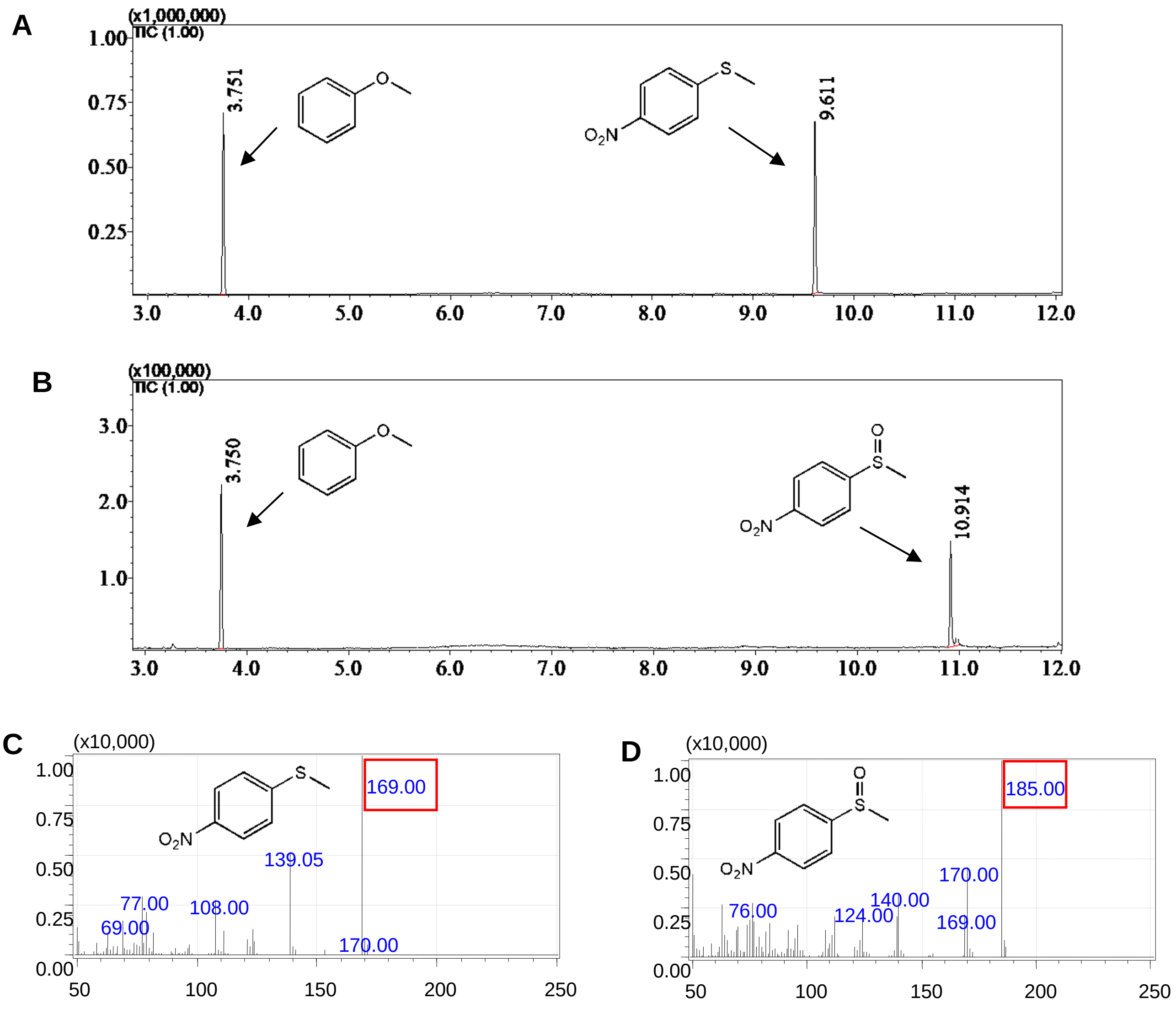

Figure S6: GC-MS TIC chromatogram of *p*NTA sulfoxidation: A) before addition of peroxide and B) after reaction completion. Peak eluted at Rt= 3.75 min is relative to anisole (internal standard). C) and D) are the EI-MS spectra of peaks eluted at Rt = 9.61 min and 10.91 min, which were identified respectively as *p*NTA and corresponding sulfoxide. Molecular ions are labeled in red.

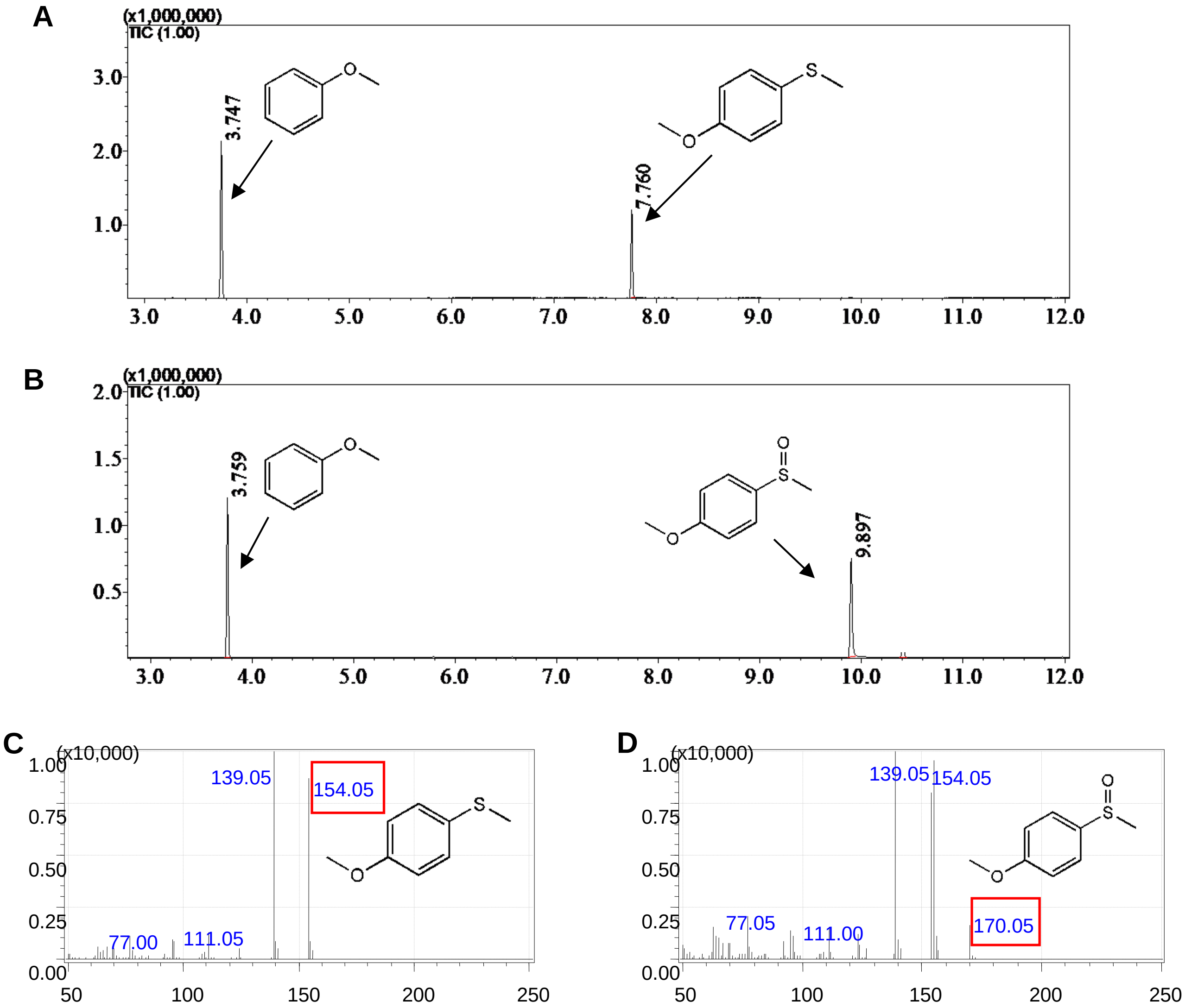

Figure S7: GC-MS TIC chromatogram of *p*MTA sulfoxidation: A) before addition of peroxide and B) after reaction completion. Peak eluted at Rt= 3.75 min is relative to anisole (internal standard). C) and D) are the EI-MS spectra of peaks eluted at Rt = 7.76 min and 9.90 min, which were identified respectively as *p*MTA and corresponding sulfoxide. Molecular ions are labeled in red.

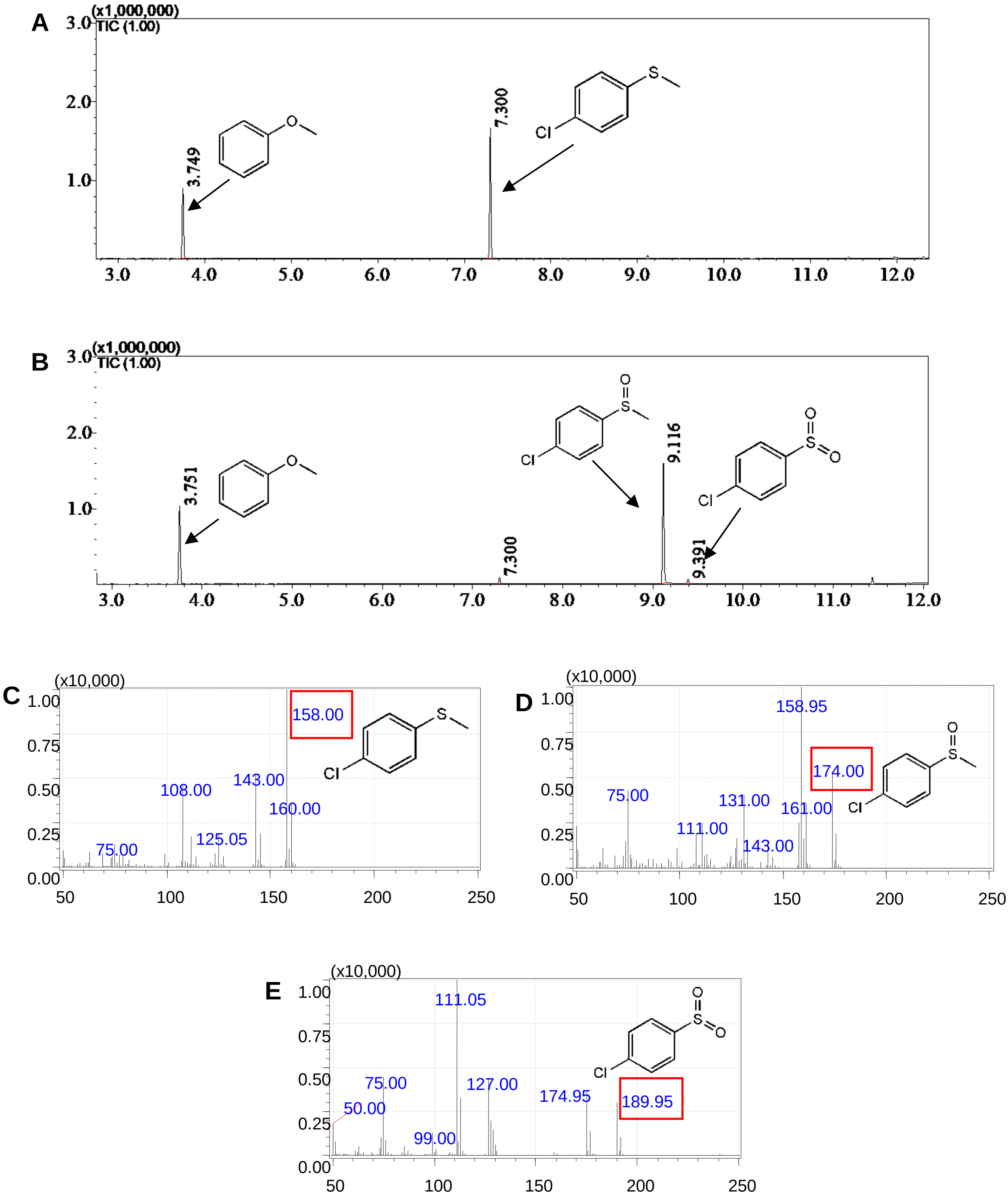

Figure S8: GC-MS TIC chromatogram of *p*CTA sulfoxidation: A) before addition of peroxide and B) after reaction completion. Peak eluted at Rt= 3.75 min is relative to anisole (internal standard). C), D) and E) are the EI-MS spectra of peaks eluted at Rt = 7.30 min, 9.12 min, and 9.39 min which were identified respectively as *p*CTA, corresponding sulfoxide and sulfone. Molecular ions are labeled in red.

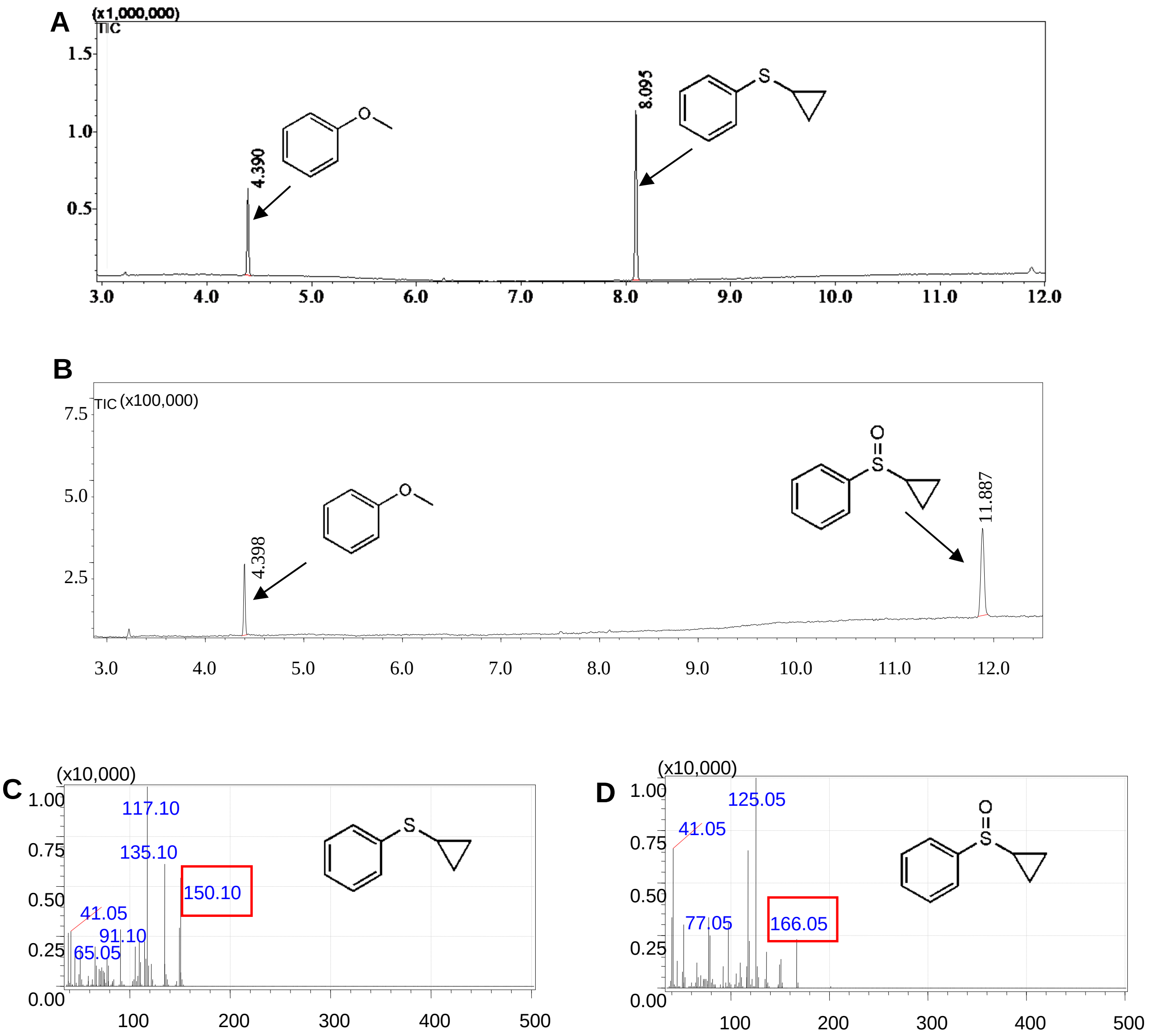

Figure S9: GC-MS TIC chromatogram of CPPS oxidation: A) before addition of peroxide and B) after reaction completion. Peak eluted at Rt= 4.40 min is relative to anisole (internal standard). C) and D) are the EI-MS spectra of peaks eluted at Rt = 8.10 min and 11.89 min which were identified respectively as CPPS and corresponding sulfoxide. Molecular ions are labeled in red.

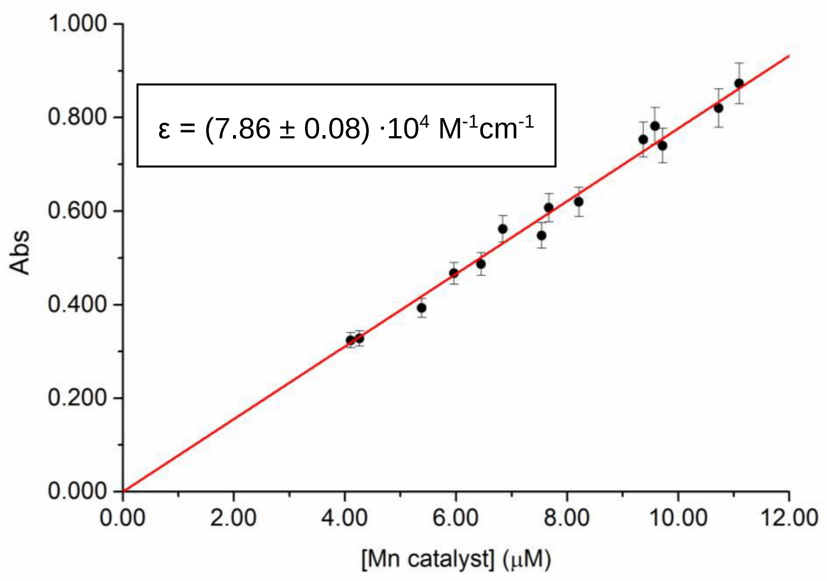

Figure S10: Plot of the absorbance at 365 nm as a function of Mn^{III}-MC6*a concentration. Data were fitted to the Lambert-Beer's law.

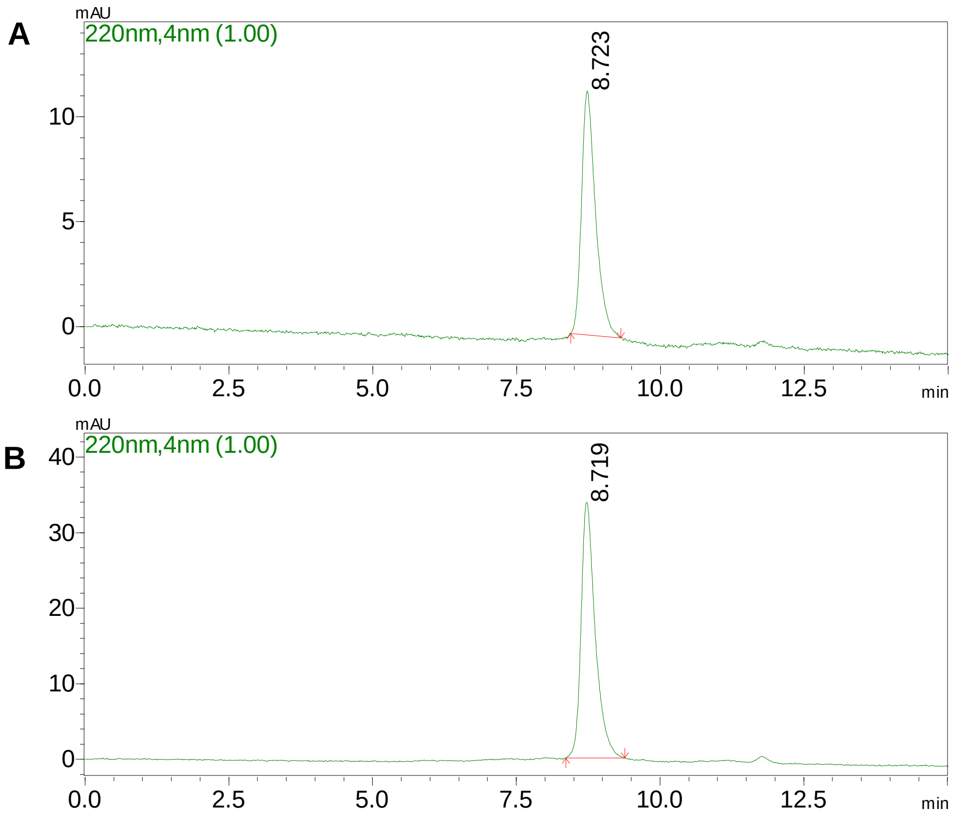

Figure S11: GFC chromatograms of: A) Mn^{III}-HRP; B) Fe^{III}-HRP (C = 0.1 mg/ml, 0.05 M sodium phosphate, 0.3 M NaCl, pH 6.8).

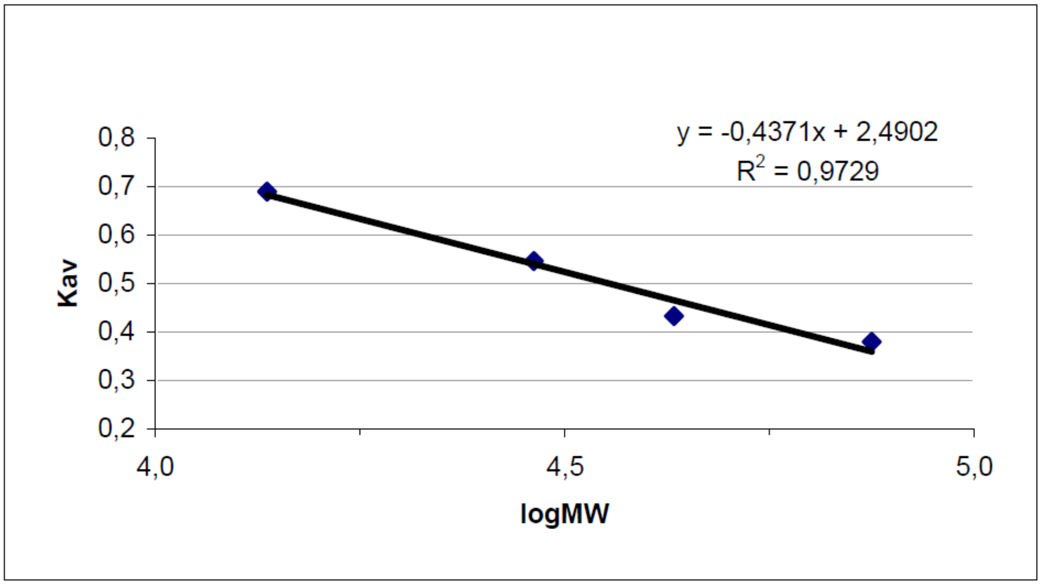

Figure S12: GFC calibration curve prepared used Conalbumin (MW: 75 kDa), Ovalbumin (MW: 43 kDa), Carbonic Anhydrase (MW: 29 kDa), Ribonuclease A (MW: 13.7 kDa).
